# Pan-cancer analysis shows that BCAP31 is a potential prognostic and immunotherapeutic biomarker for multiple cancer types

**DOI:** 10.3389/fimmu.2024.1507375

**Published:** 2024-12-16

**Authors:** Yangyong Sun, Zhi Li, Jianchao Liu, Ying Xiao, Yaqiang Pan, Benbo Lv, Xufeng Wang, Zhiqiang Lin

**Affiliations:** ^1^ Department of Cardiothoracic Surgery, Affiliated People’s Hospital of Jiangsu University, Zhenjiang, Jiangsu, China; ^2^ Department of Emergency, Nanjing Jiangning Hospital, Nanjing, Jiangsu, China; ^3^ Department of Otolaryngology, the Affiliated Suzhou Hospital of Nanjing Medical University, Suzhou Municipal Hospital, Gusu School, Nanjing Medical University, Suzhou, Jiangsu, China

**Keywords:** BCAP31, prognosis, biomarker, target therapy, pan-cancer

## Abstract

**Background:**

B-cell receptor-associated protein 31 (BCAP31) is a widely expressed transmembrane protein primarily located in the endoplasmic reticulum (ER), including the ER-mitochondria associated membranes. Emerging evidence suggests that BCAP31 may play a role in cancer development and progression, although its specific effects across different cancer types remain incompletely understood.

**Methods:**

The raw data on BCAP31 expression in tumor and adjacent non-tumor (paracancerous) samples were obtained from the Broad Institute Cancer Cell Line Encyclopedia (CCLE) and UCSC databases. We also examined the association between BCAP31 expression and clinicopathological factors. Using the Cox proportional hazards model, we found that high BCAP31 levels were linked to poor prognosis. To further explore BCAP31’s role, we analyzed the relationship between copy number variations (CNV) and BCAP31 mRNA expression using data from The Cancer Genome Atlas (TCGA). Additionally, the association between BCAP31 expression and signature pathway scores from the MsigDB database provided insights into the tumor biology and immunological characteristics of BCAP31.We assessed the relationship between tumor immune infiltration and BCAP31 expression using the TIMER2 and ImmuCellAI databases. The ESTIMATE computational method was employed to estimate the proportion of immune cells infiltrating the tumors, as well as the stromal and immune components, based on TCGA data. To investigate drug sensitivity in relation to BCAP31 expression, we utilized GDSC2 data, which included responses to 198 medications. We explored the relationship between BCAP31 gene expression and response to immunotherapy. Additionally, the study involved culturing KYSE-150 cells under standard conditions and using siRNA-mediated knockdown of BCAP31 to assess its function. Key experiments included Western blotting (WB) to confirm BCAP31 knockdown, MTT assays for cell proliferation, colony formation assays for growth potential, Transwell assays for migration and invasion, and wound healing assays for motility. Additionally, immunohistochemistry (IHC) was performed on tumor and adjacent normal tissue samples to evaluate BCAP31 expression levels.

**Results:**

BCAP31 was found to be significantly overexpressed in several prevalent malignancies and was associated with poor prognosis. Cox regression analysis across all cancer types revealed that higher BCAP31 levels were predominantly linked to worse overall survival (OS), disease-free interval (DFI), disease-specific survival (DSS), and progression-free interval (PFI). In most malignancies, increased BCAP31 expression was positively correlated with higher CNV. Additionally, BCAP31 expression was strongly associated with the tumor microenvironment (TME), influencing the levels of infiltrating immune cells, immune-related genes, and immune-related pathways. Drug sensitivity analysis identified six medications that showed a significant positive correlation with BCAP31 expression. Furthermore, BCAP31 expression impacted the outcomes and prognosis of cancer patients undergoing immune therapy. The functional assays demonstrated that BCAP31 knockdown in KYSE-150 cells significantly inhibited cell migration, invasion, and proliferation while enhancing colony formation ability. WB and immunohistochemistry analyses confirmed elevated BCAP31 expression in tumor tissues compared to adjacent normal tissues in esophageal cancer, lung adenocarcinoma, and gastric adenocarcinoma.

**Conclusion:**

BCAP31 has the potential to serve as a biomarker for cancer immunology, particularly in relation to immune cell infiltration, and as an indicator of poor prognosis. These findings provide a new perspective that could inform the development of more targeted cancer therapy strategies.

## Introduction

The prevalence and fatality rate of cancer is consistently rising, resulting in cancer being the second most prominent cause of mortality globally. This alarming trend poses a significant risk to human well-being while also placing substantial healthcare and economic strain on society (1). Cancer is a pathological condition characterized by dysregulation across several biological levels, known as omics, and fundamental traits of cancer cells include sustained proliferation and mitosis. These traits are facilitated by the stimulation of growth signals that exert influence on the development and advancement of cancer (2).

B-cell receptor-associated protein 31 (BCAP31) was first identified and characterized by the research team led by Michael Reth in the year 1994 (3). The nomenclature of this entity was derived from its co-purification with immunoglobulin D, a component of B-cell receptors, as well as its manifestation of an observable molecular weight of 31 kDa on electrophoretic gels under denaturing conditions. BAP31, a transmembrane protein, has been promptly recognized as having widespread expression and mostly localizing in the Endoplasmic Reticulum (ER). Its primary roles in this cellular compartment include acting as a specific transmembrane protein chaperone as well as modulating apoptosis (4, 5).

Previous studies have shown an elevation of BCAP31 in several types of cancer, including cervical cancer (6), colorectal cancer (7), hepatocellular carcinoma (8), non-small lung cancer (9), gastric intestinal-type adenocarcinoma (10), as well as malignant melanoma (11). This elevation has been associated with a poor prognosis. It has been observed that low expression regarding BCAP31 corresponds with poor prognosis in colorectal cancer and hepatocellular carcinoma (12, 13). Furthermore, the act of eliminating it in U2OS osteosarcoma cells resulted in increased tumorigenicity both *in-vitro* as well as in a xenograft model. This effect was related to its role in suppressing autophagy (14). It was indicated that BCAP31 possesses the ability to function as a prognostic indicator for several types of malignancies.

Furthermore, it is noteworthy to mention that much research has shown an unexpected immunotherapy impact of BCAP31 on many types of malignancies. In the first stages of the study, it was discovered that the use of an immunotoxin that consists of Pseudomonas exotoxin A conjugated to a fragment of an anti-BAP31 antibody showed efficacy in inducing cell death in MDA-231 breast cancer cells when tested in a controlled laboratory setting (15). In a recent study, it was shown that vaccine immunotherapy utilizing BAP31-encoding plasmid resulted in a robust immunogenic reaction. This reaction effectively inhibited the development of tumors in a mouse model specifically designed to mimic malignant melanoma (11). Moreover, it has been demonstrated that the production of an intracellular antibody fragment against BAP31, known as an intrabody, effectively hinders the interaction with p27Kip1 and consequently inhibits the growth of mouse gastric cancer tumor xenografts. This inhibition was observed without any observable adverse effects (10). Additionally, the administration of the BAP31 antibody through interperitoneal injections demonstrated similar efficacy in limiting the development with regard to mouse hepatocellular carcinoma xenografts (8). Moreover, there is an increasing inclination towards using microRNAs for medicinal purposes (16). Prior research has demonstrated miR-362’s effectiveness in suppressing cancer development in mouse tumor xenografts via its targeting of BAP31 following transfection (17).

Nevertheless, most studies that have looked into how BCAP31 functions in malignancies thus far have only looked at a specific type of malignancy. There has not been a pan-cancer investigation into the relationship between BCAP31 and various tumors. As a result, we examined BCAP31 expression levels and their associations with prognosis in various malignancies using a number of databases, including UCSC, CCLE, as well as cBioPortal. Additionally, we examined potential relationships between BCAP31 expression and the Tumor Mutational Burden (TMB), copy number mutation, as well as immune infiltration in 33 distinct cancer types. We also performed enrichment analysis as well as co-expression studies with respect to immune-related genes with BCAP31 to investigate the biological roles of BCAP31 in malignancies. By influencing tumor-infiltrating immune cells and TMB, the outcomes we discovered demonstrate that BCAP31 can be utilized as a prognostic factor for several malignancies as well as can be a key player in tumor immunity. This investigation can shed light on BCAP31’s function in tumor immunotherapy.

## Materials and methods

### Data collection

Utilizing the UCSC Xena platform, the RNA-sequencing samples derived from the Genotype-Tissue Expression (GTEx) as well as TCGA datasets were acquired (18). The CCLE database served as the source of information regarding every tumor cell line. Subsequently, the expression levels of these cell lines across 21 different tissues were examined and assessed based on their tissue origins. The data pertaining to the cohort undergoing immunotherapy was obtained by accessing the **Supplementary Materials** of a previously published study (19–21).

### Cox regression analysis and survival analysis

Through the R software, Cox regression analysis was performed to investigate the relationship between BCAP31 expression as well as the disease-specific survival (DSS), overall survival (OS) and disease-free interval (DFI), in addition to the progression-free interval (PFI) of patients for various cancer types. The research utilized the The Cancer Genome Atlas (TCGA) datasets. Patients were divided into two groups based on BCAP31 expression levels using the median value as the threshold. The R packages “survival” as well as “survminer” were used to create the survival curves. We also looked at the relationship between BCAP31 expression as well as OS in a pan-cancer cohort using the R package “forestplot”.

### Analysis of CNA in BCAP31

The identification of copy number variations (CNVs) of BCAP31 in pan-cancer patients was conducted via the cBioPortal for Cancer Genomics platform, accessible at http://www.cbioportal.org/.

### Tumor mutant burden

The mutation data was acquired from the UCSC Xena. Subsequently, it was then processed using the Pearl programming language for extraction. Subsequently, we conducted an examination and integration of mutant data using the “maftools” software (22), followed by an analysis of the disparities in TMB between the BCAP31 high-risk as well as low-risk cohorts. esophageal squamous cell carcinoma (ESCA) samples were stratified into high and low groups based on the median expression level of BCAP31. Logistic regression was then employed to investigate the association between tumor mutation burden and ESCA cohort.

### Gene set variation analysis

The use of GSVA was performed in order to examine the probable biological mechanism associated with BCAP31 across various types of cancer. The Molecular Signatures Database (Msigdb) provided the gene set for the Hallmark pathway (23). The R language “GSVA” package (24) was employed to determine the Hallmark pathway scores for every type of malignancy.

### Immune feature analysis

The StromalScore, ImmunoScore, ESTIMATEScore, and TumorPurity scores have been generated using the ESTIMATE (25). Additionally, we used the approach proposed by Zeng et al. (26) to evaluate the TME’s biological mechanisms, including those connected to the immune system. Utilizing the Immune Cell Abundance Identifier (ImmuCellAI) as well as Tumor Immune Estimation Resource 2.0 (TIMER2.0) databases, the relationship between BCAP31 and immune cell infiltration was examined. Additionally, spearman test is utilized to examine the relationship between BCAP31 expression and immune-associated genes, for instance, Major Histocompatibility (MHC), chemokine-receptor, chemokine, in addition to immune-activating genes.

### Drug sensitivity analysis

Next, we investigated the correlation between BCAP31 expression as well as drug sensitivity using data obtained from the GDSC2 database. Following this, the relationship between medicines as well as the level of BCAP31 expression was investigated using spearman correlation analysis. The drugs exhibiting the highest six positive correlations were then presented.

### Cell culture

KYSE-150 cell was obtained from Wuhan Punosai Life Technology Co., Ltd. KYSE-150 cell was grown in Roswell Park Memorial Institute-1640 (RPMI-1640) medium supplemented with 10% FBS and 1% streptomycin and penicillin. During cultivation at 37°C and 5.0% CO2, the KYSE-150 cell was frequently examined for mycoplasma contamination.

### Human specimens

The study comprised a total of 5 tissue samples of ESCA and 5 corresponding paracancerous samples, 5 samples of lung adenocarcinoma (LUAD) and 5 corresponding paracancerous samples, and 5 samples of gastric adenocarcinoma (GA) and 5 corresponding paracancerous samples. All samples were collected from individuals who underwent surgery at the Affiliated People’s Hospital of Jiangsu University between April 2023 and September 2023. All procedures were conducted in accordance with relevant rules and regulations. The study and experimental procedures were approved by the Ethics Committee of the Affiliated People’s Hospital of Jiangsu University (Ethics Number: K-20230079-W), and informed consent was obtained from the patients.

### Knockdown assay, cell transfection

To generate BCAP31-knockdown KYSE-150 cells, small interfering RNA (siRNA) targeting BCAP31 (si-BCAP31) and a negative control siRNA (si-NC) were synthesized by Shanghai Jima Biotechnology Co., Ltd. Three knockdown sequences were constructed and transfected into KYSE-150 cells using Polyplus jetPRIME reagent (Polyplus, Illkirch, France) via liposome transfection. Transfection efficiency was verified by Western blot 48 hours post-transfection. All experiments were performed in triplicate, including both the BCAP31 knockdown group (si-BCAP31) and the negative control group (si-NC), to ensure statistical reliability.

### Western blot analysis

The KYSE-150 cells were washed three times with ice-cold PBS and then lysed in RIPA buffer for 30 minutes at 4°C. Protein concentrations in the supernatants were determined using a bicinchoninic acid (BCA) protein assay kit after the lysates were centrifuged. Protein concentrations were then adjusted using lysis buffer. The samples were mixed with protein loading buffer and heated at 100°C for 10 minutes. Equal amounts of protein from all groups were separated on 12.5% SDS-PAGE and then transferred to PVDF membranes. The membranes were blocked at room temperature for 2 hours with 5% non-fat milk, followed by overnight incubation at 4°C with primary antibodies against BCAP31 and β-actin, each at a 1:1000 dilution. After three washes with TBST, the membranes were incubated with HRP-conjugated goat anti-rabbit or anti-mouse IgG for 2 hours at room temperature. Protein bands were then detected and quantified using ImageJ software.

### MTT assay

KYSE-150 cells were seeded into 96-well plates at a density of 1×10^4^ cells per well in 100 μL of cell growth medium. Each group was organized into six replicate wells and incubated at 37°C in an incubator with 5% CO2. After the incubation period, 10 μL of MTT solution was added to each well, followed by an additional 4 hours of incubation. After horizontal centrifugation, the supernatant was carefully removed, and 150 μL of dimethyl sulfoxide (DMSO) was added to each well. The plates were then gently agitated for 10 minutes, and the optical density at 490 nm was measured using a microplate reader.

### Colony formation assay

Each group of KYSE-150 cells (si-BCAP31 and si-NC) was seeded in 6-well plates (500 cells per well) and cultured for 2 weeks to allow colony formation. Colonies were fixed with paraformaldehyde and stained with 0.4% crystal violet, making them visible for counting. Colony formation was assessed in triplicate wells for each group, and the entire experiment was independently repeated three times to confirm reproducibility.

### Transwell assay

The Transwell migration and invasion assays were conducted using 24-well plates with Transwell inserts. Cells were counted and resuspended in serum-free RPMI-1640 medium at a concentration of 5×10^4^ cells/mL. A 200 μL aliquot of the cell suspension was added to the upper chamber of the Transwell, while the lower chamber was filled with RPMI-1640 medium supplemented with 10% FBS as a chemoattractant. Both the BCAP31 knockdown group (si-BCAP31) and the negative control group (si-NC) were set up with triplicate wells for each experimental condition. Following 24 hours of incubation at 37°C in 5% CO_2_, migrated cells were fixed, stained with 0.1% crystal violet, and quantified. Images were captured from six randomly selected fields per well using an inverted microscope, and the experiment was independently repeated three times for consistency.

### Wound healing assay

The studies were conducted according to the manufacturer’s instructions. Briefly, cells were seeded onto 24-well culture plates at a density of 2.0×10^5^ cells per well and incubated at 37°C with 5% CO2 until they formed a monolayer. Once the monolayer was established, a scratch was made using a 1000 μL pipette tip, followed by two rinses with PBS. The medium was then replaced with RPMI-1640, and the cells were further incubated at 37°C with 5% CO2.

### Immunohistochemistry

To reduce endogenous peroxidase activity, paraffin-embedded ESCA tissues were dewaxed and incubated in 3% hydrogen peroxide at room temperature for 30 minutes. Antigen retrieval was performed by steam-heating the tissues in citrate buffer for 30 minutes. The tissues were then treated with 5% bovine serum albumin to block non-specific binding. Afterward, the BCAP31 antibody (1:200) was added, and the tissues were incubated overnight at 4°C. The following day, a secondary antibody was applied, and the tissues were incubated at 37°C for 30 minutes. Subsequently, the StreptAvidin Biotin Complex was added and incubated for 30 minutes at 37°C. After resin sealing, the tissue sections were treated with a diaminobenzidine substrate and counterstained with hematoxylin for microscopic examination.

### Statistics analysis

The Statistical Product and Service Solutions (SPSS) software was used to perform the statistical calculations for the molecular biology verification. A two-tailed student’s t-test was the statistical analysis employed in the present investigation to evaluate and compare the differences between the two groups. To ascertain the statistical significance across numerous groups, the One-factor Analysis of Variance was employed. The data are presented in the form of means ± Standard Deviation (SD). Any difference with a P<0.05 was considered statistically significant.

## Results

### The expression profiles of BCAP31

**Figure 1:**
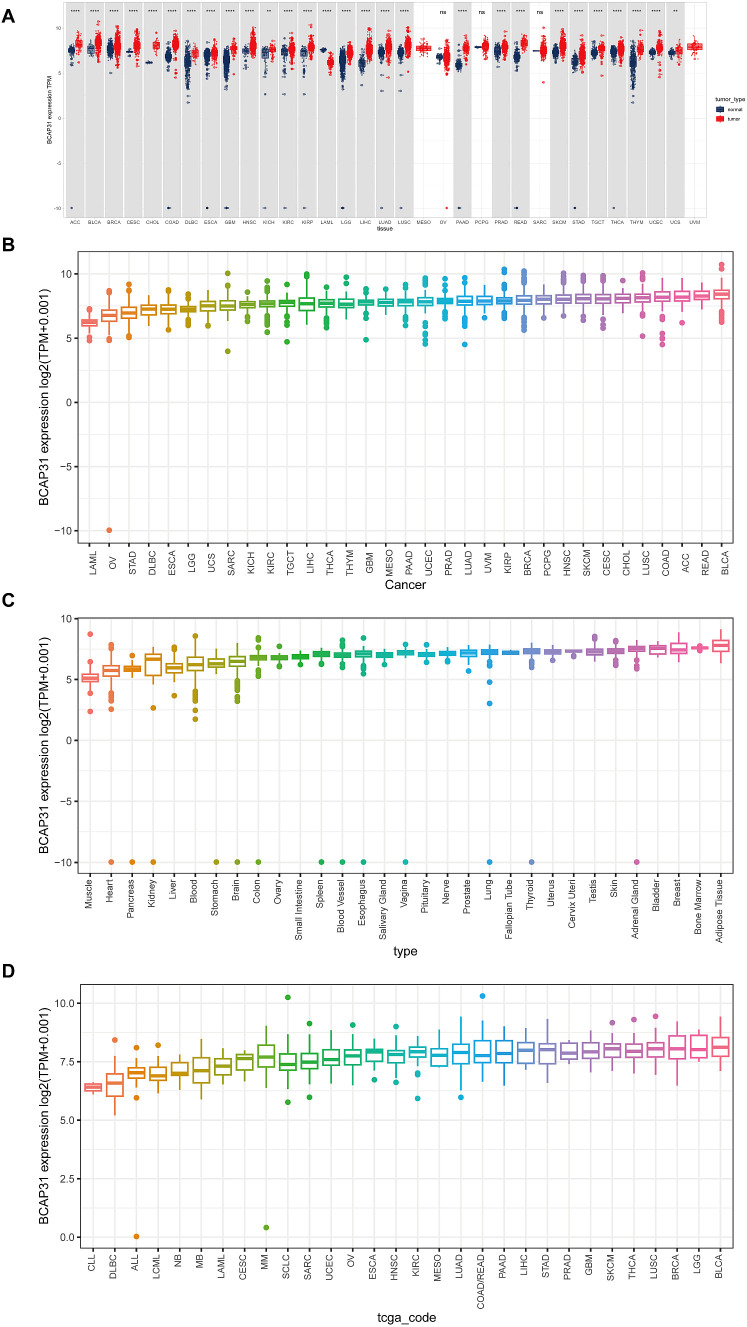
Differential expression of BCAP31 gene across various datasets. **(A)** Differential expression of the BCAP31 gene between tumor (red) and normal (blue) tissues across multiple tissue types. The y-axis represents log2(TPM + 0.001) values, with each box representing the distribution of expression levels for each cancer type. **p < 0.01, ****p < 0.0001, ns = not significant. **(B)** Expression boxplot of BCAP31 gene across different cancer types in the TCGA dataset. The y-axis shows log2(TPM + 0.001) values, with each box representing the distribution of expression levels for each cancer type. **(C)** Expression boxplot of BCAP31 gene across various tissue types in the GTEx dataset. The y-axis shows log2(TPM + 0.001) values, illustrating the expression distribution across normal tissues from different organs. **(D)** Expression boxplot of BCAP31 gene across different cancer cell lines in the CCLE dataset. The y-axis shows log2(TPM + 0.001) values, with each box representing the distribution of expression levels in specific cell lines.

BCAP31 expression in different tumors as well as normal tissues was determined by analyzing mRNA data obtained from the UCSC Xena and CCLE databases. Based on the analysis of the UCSC Xena dataset, it was observed that BCAP31 exhibited differential expression across various tumor types. Specifically, high expression levels were detected in certain tumors, while low expression levels were observed in others. Notably, this pattern was not observed in mesothelioma (MESO), ovarian Serous cystadenocarcinoma (OV), sarcoma (SARC), pheochromocytoma and paraganglioma (PCPG), as well as uveal melanoma (UVM) (**Figure 1A**). These findings were generally consistent with the results obtained from the TCGA dataset (**Figure 1B**). The expression pattern of BCAP31 in GTEx cohorts was shown in **Figure 1C**, revealing a progressive rise in BCAP31 expression from the left to the right. Additional analysis of several cancer cell lines revealed that adipose tissue had the greatest level of gene expression, while muscle tissue exhibited the lowest level of gene expression. Additionally, an investigation was conducted on the BCAP31 protein expression via the CCLE database. The BCAP31 expression in cancer cells exhibited a five-fold rise, as seen in **Figure 1D**.

### Expression of BCAP31 in paired samples of 12 tumors in TCGA database

**Figure 2:**
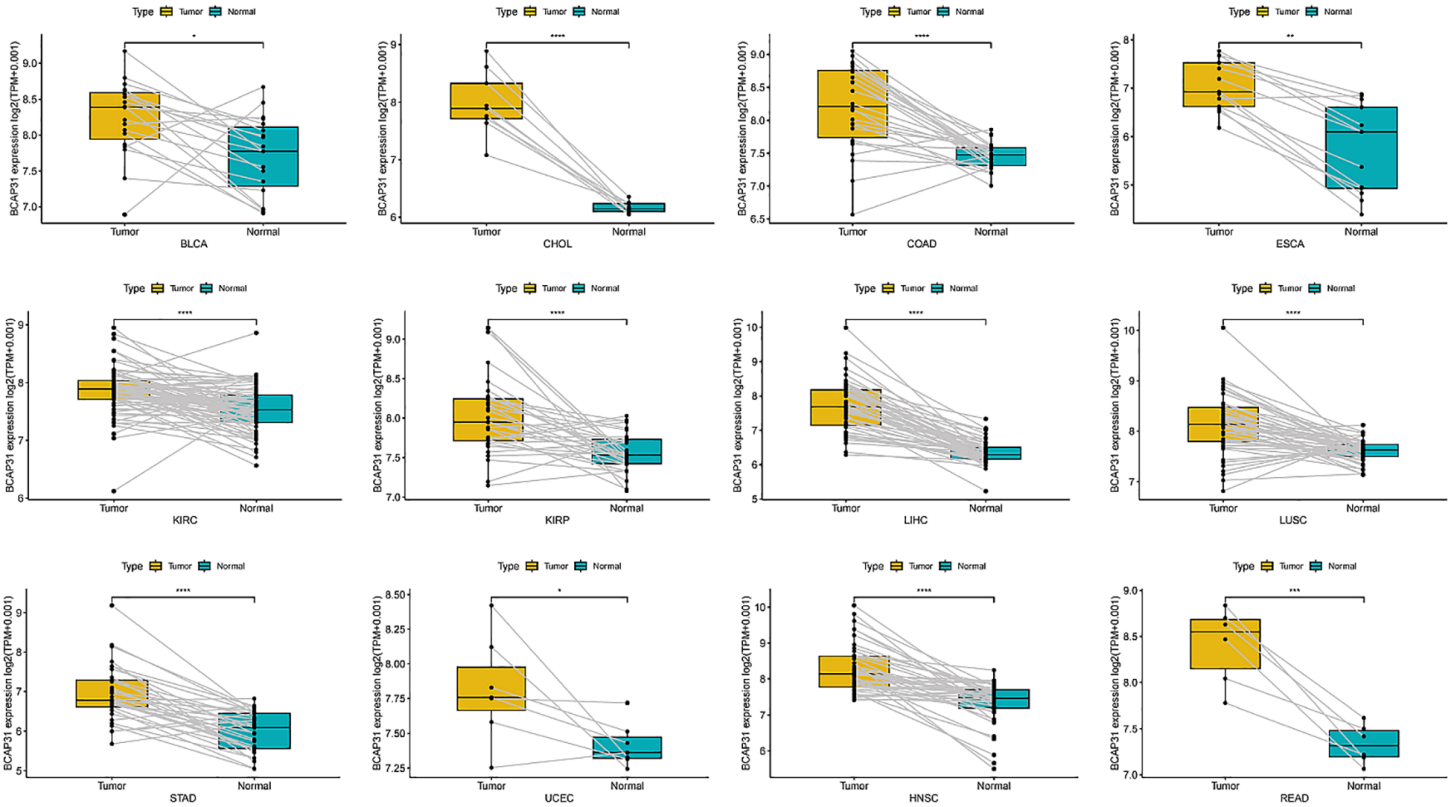
Differential expression of BCAP31 gene in paired tumor and adjacent normal tissues across various cancer types. Boxplots depict the log2(TPM + 0.001) expression values of BCAP31 in tumor (yellow) and matched adjacent normal (cyan) tissues. BLCA (Bladder Urothelial Carcinoma); CHOL (Cholangiocarcinoma); COAD (Colon Adenocarcinoma); ESCA (Esophageal Carcinoma); KIRC (Kidney Renal Clear Cell Carcinoma); KIRP (Kidney Renal Papillary Cell Carcinoma); LIHC (Liver Hepatocellular Carcinoma); LUSC (Lung Squamous Cell Carcinoma); STAD (Stomach Adenocarcinoma); UCEC (Uterine Corpus Endometrial Carcinoma); HNSC (Head and Neck Squamous Cell Carcinoma); READ (Rectum Adenocarcinoma). *p < 0.05, **p < 0.01, ***p < 0.001, ****p < 0.0001. Paired data points are connected by lines to show individual sample comparisons.

An analysis was conducted to examine BCAP31 expression across 33 distinct cancer types using TCGA-matched data. Significant differences in BCAP31 expression were observed in cholangiocarcinoma (CHOL), esca, bladder urothelial carcinoma (BLCA), kidney renal clear cell carcinoma (KIRC), adrenocortical carcinoma (ACC), colon adenocarcinoma (COAD), lung squamous cell carcinoma (LUSC), kidney renal papillary cell carcinoma (KIRP), liver hepatocellular carcinoma (LIHC), stomach adenocarcinoma (STAD), uterine corpus endometrial carcinoma (UCEC), head and neck squamous cell carcinoma (HNSC), and rectum adenocarcinoma (READ) (**Figures 2A–L**). The corresponding paired tissue sample numbers for these analyses were 19, 9, 26, 13, 72, 32, 50, 50, 33, 7, 43, and 6, respectively.

### The correlation between BCAP31 expression and clinicopathology

**Figure 3:**
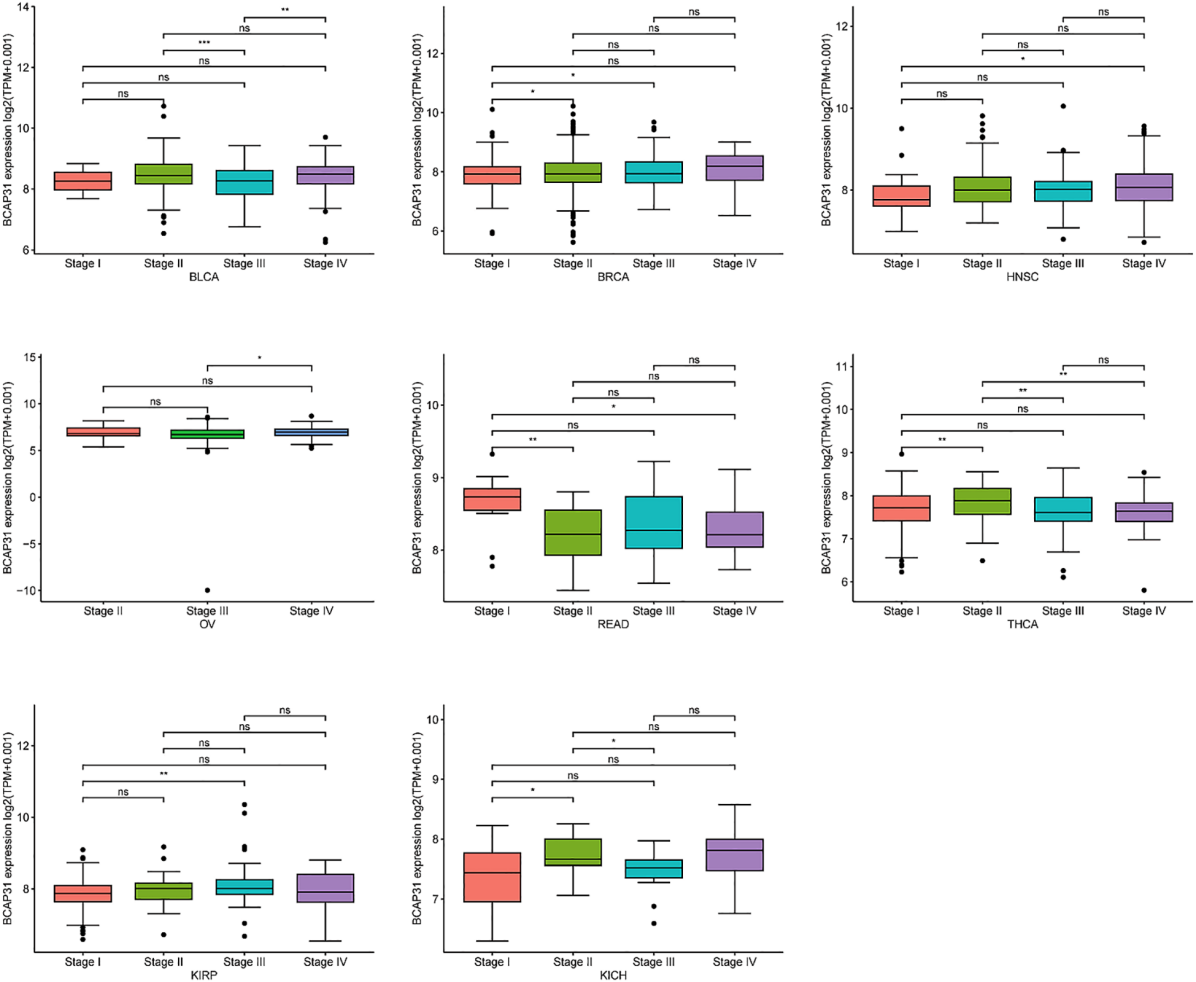
The expression of BCAP31 across different cancer stages in pan-cancer analysis. Boxplots show the log2(TPM + 0.001) expression levels of BCAP31 in various stages (Stage I, II, III, and IV) across different cancer types. BLCA (Bladder Urothelial Carcinoma); BRCA (Breast Invasive Carcinoma); HNSC (Head and Neck Squamous Cell Carcinoma); OV (Ovarian Serous Cystadenocarcinoma); READ (Rectum Adenocarcinoma); THCA (Thyroid Carcinoma); KIRP (Kidney Renal Papillary Cell Carcinoma); KICH (Kidney Chromophobe). *p < 0.05, **p < 0.01, ***p < 0.001, and “ns” for non-significant comparisons.

We investigated the relationship between BCAP31 expression and tumor stage across several cancer types, ranging from stage I to stage IV. Our results indicated a significant correlation between BCAP31 expression and tumor stage in specific cancers, including BLCA, breast invasive carcinoma (BRCA), HNSC, OV, READ, thyroid carcinoma (THCA), KIRP, and kidney chromophobe (KICH). Notably, in some cancer types, BCAP31 expression varied significantly between earlier and later stages, suggesting a potential role for BCAP31 in tumor progression (**Figure 3**).

### Prognostic value of BCAP31

**Figure 4:**
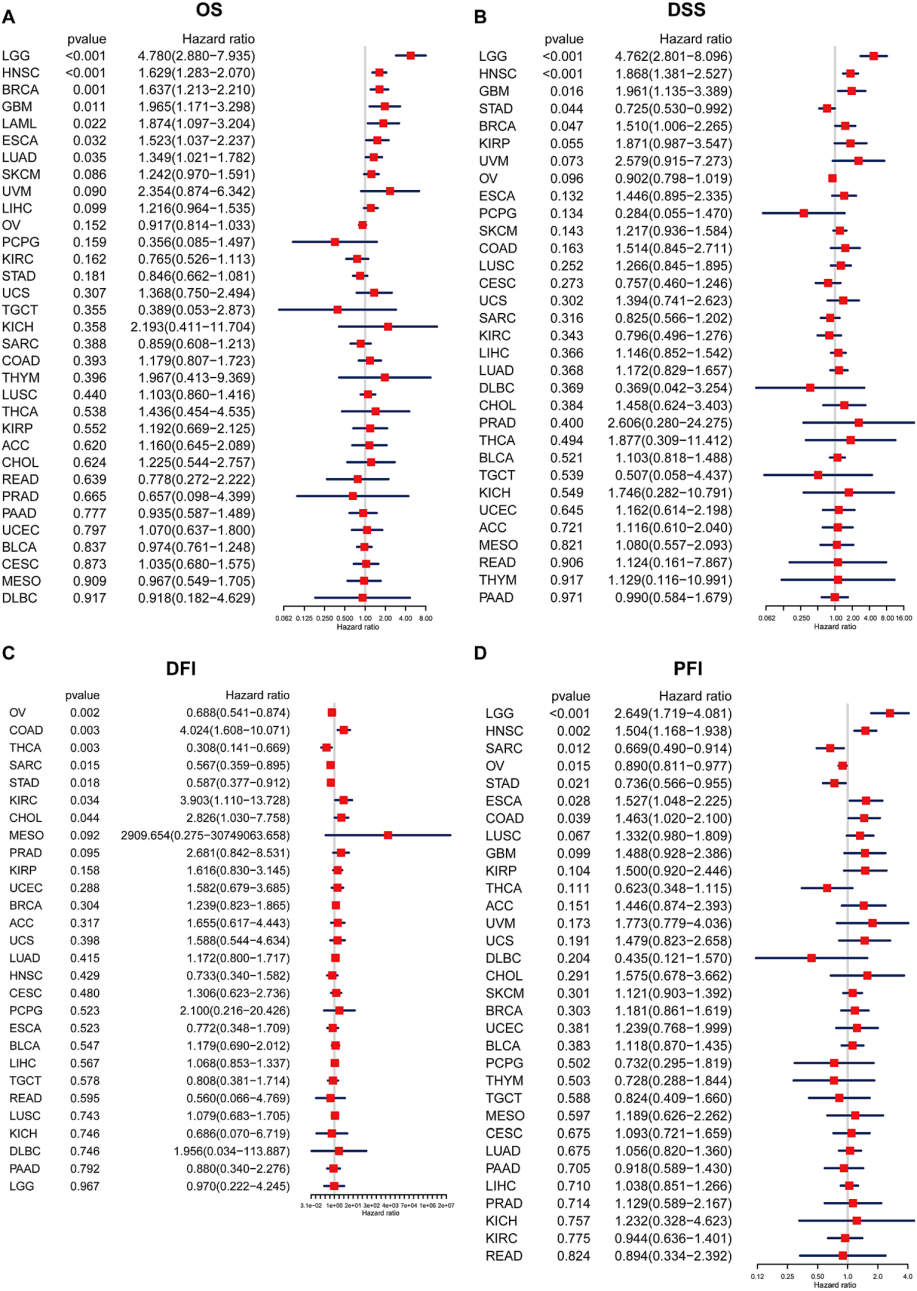
Association between BCAP31 expression levels and clinical outcomes in various cancer types. Forest plots illustrate the hazard ratios (HR) and p-values for the association of BCAP31 expression with four clinical outcomes. **(A)** OS (Overall Survival); **(B)** DSS (Disease-Specific Survival); **(C)** DFI (Disease-Free Interval); **(D)** PFI (Progression-Free Interval). The hazard ratio (HR) and its 95% confidence interval are presented for different cancer types. An HR > 1 indicates that higher BCAP31 expression is associated with an increased risk, while an HR < 1 suggests a protective effect. Red squares represent the HR values, with the horizontal blue lines indicating the 95% confidence intervals. Significant associations are highlighted based on the p-values provided.

In order to examine the relationship between the BCAP31 expression level and prognosis, we used OS, DFI, DSS, as well as PFI as indicators to assess the prognostic implications of BCAP31 in several cancer types. The BCAP31 expression was demonstrated to be substantially linked with poor OS in many cancer types, including brain lower grade glioma (BLGG) (p < 0.001), HNSC (p < 0.001), BRCA (p = 0.001), glioblastoma multiforme (GBM) (p < 0.011), acute myeloid leukemia (LAML) (p = 0.022), ESCA (p = 0.032), and LUAD (p = 0.035) (**Figure 4A**). Pertaining to DSS, it was shown that greater expression with respect to BCAP31 was linked with shorter DSS in LGG (p < 0.001), HNSC (p < 0.001), GBM (p = 0.016), and BRCA (p = 0.047). In contrast, the downregulation of BCAP31 was shown to be associated with extended DSS in patients with STAD (p = 0.044), as seen in **Figure 4B**. Furthermore, the examination of DFI data demonstrated correlations between elevated BCAP31 expression as well as unfavorable prognosis in individuals diagnosed with COAD (p = 0.003), KIRC (p = 0.034), and CHOL (p = 0.044). Conversely, in patients with OV (p = 0.002), THCA (p = 0.003), SARC (p = 0.015), as well as STAD (p = 0.018), BCAP31 expression displayed a contrasting association with prognosis (**Figure 4C**). Lastly, the findings revealed that the overexpression of BCAP31 was associated with a significantly shorter PFI in patients with LGG (p < 0.001), HNSC (p = 0.002), ESCA (p = 0.028), and COAD (p = 0.039). On the contrary, the downregulation of BCAP31 was associated with a significant PFI in SARC (p = 0.012), OV (p = 0.015), and STAD (p < 0.021), as seen in **Figure 4D**.

### Survival analysis

**Figure 5:**
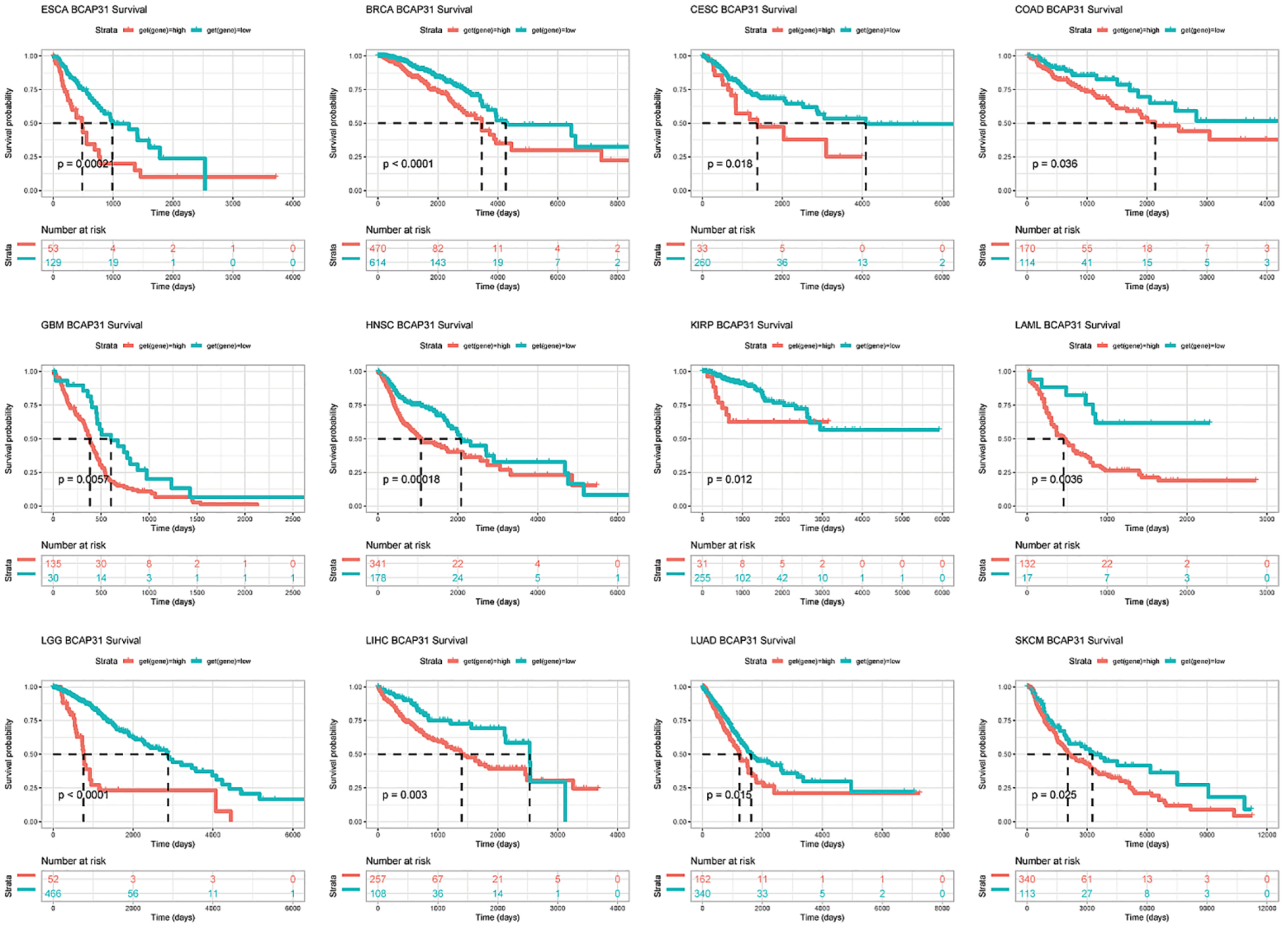
Kaplan-Meier survival curves for BCAP31 expression in various cancer types. Kaplan-Meier survival analysis was performed to assess the impact of high (red) versus low (blue) BCAP31 expression levels on overall survival across different cancer types. ESCA (Esophageal Carcinoma), p = 0.0002; BRCA (Breast Invasive Carcinoma), p < 0.0001; CESC (Cervical Squamous Cell Carcinoma and Endocervical Adenocarcinoma), p = 0.018; ;COAD (Colon Adenocarcinoma), p = 0.036; GBM (Glioblastoma Multiforme), p = 0.005; HNSC (Head and Neck Squamous Cell Carcinoma), p = 0.0018; KIRP (Kidney Renal Papillary Cell Carcinoma), p = 0.012; LAML (Acute Myeloid Leukemia), p = 0.0036; LGG (Brain Lower Grade Glioma), p < 0.0001; LIHC (Liver Hepatocellular Carcinoma), p = 0.003; LUAD (Lung Adenocarcinoma), p = 0.015; SKCM (Skin Cutaneous Melanoma), p = 0.025. The x-axis represents time in days, and the y-axis represents survival probability. The Number at risk table below each plot indicates the number of patients remaining in each expression group at different time points, providing insight into survival trends based on BCAP31 expression levels.

Subsequently, a more thorough analysis of the relationship between BCAP31 expression as well as cancer prognosis was carried out utilizing the Kaplan-Meier plot. The outcomes regarding the correlation study showed a substantial relationship between BCAP31 expression as well as the prognosis of various cancer kinds. The findings indicated that the upregulation of BCAP31 expression was associated with an unfavorable prognosis in twelve distinct cancer types, namely ESCA, cervical squamous cell carcinoma and endocervical adenocarcinoma (CESC), LIHC, BRCA, COAD, HNSC, GBM, LAML, KIRP, LGG, LUAD, and SKCM. Furthermore, the findings showed that survival rates were substantially lower for those in the high BCAP31 group than those in the low BCAP31 group (**Figure 5**).

### Gene mutation analysis and tumor mutant burden analysis

**Figure 6:**
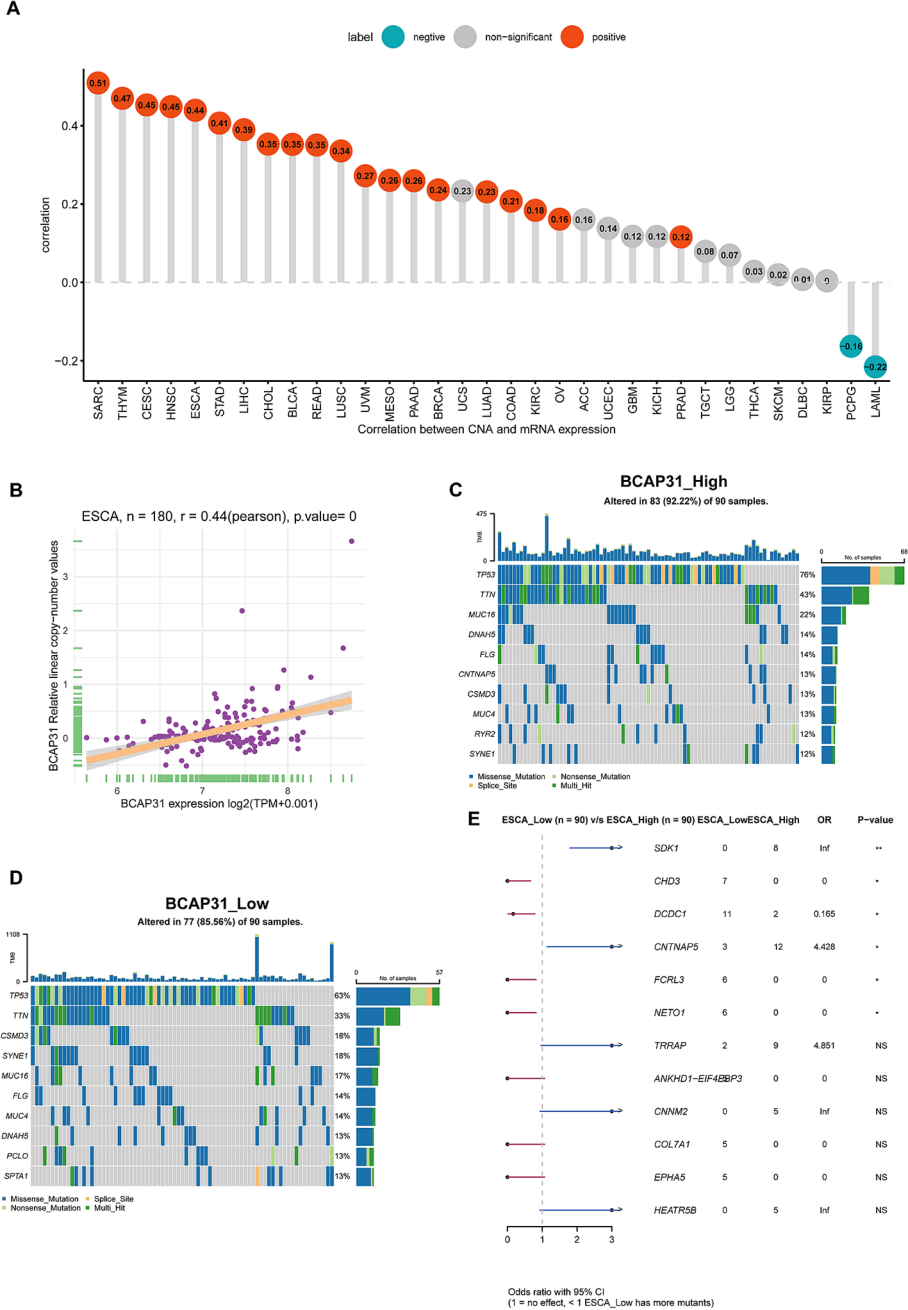
Correlations and genetic variations of BCAP31 in ESCA and pan-cancer analysis. **(A)** Bar chart showing the correlation between BCAP31 gene expression and copy number alterations (CNA) across various cancers, with green bars indicating negative correlations and red bars indicating positive correlations; **(B)** Scatter plot showing the correlation between BCAP31 expression and CNA specifically in ESCA (Esophageal Carcinoma), with a Pearson correlation coefficient of 0.44 (p < 0.001); **(C, D)** Mutation profiles of selected genes in high and low BCAP31 expression groups in ESCA, illustrating differences in mutation frequencies; **(E)** Forest plot displaying odds ratios and p-values for the comparison of gene mutation frequencies between high and low BCAP31 expression groups in ESCA. *p < 0.05, **p < 0.01. ns for non-significant comparisons.

Following this, we performed an investigation into the correlation between BCAP31 as well as CNA in pan-cancer. The research’s findings show a substantial positive correlation between BCAP31 as well as CNA in a number of cancer types, which includes SARC, CESC, HNSC, thymoma (THYM), ESCA, STAD, LIHC, LUAD, CHOL, BLCA, OV, READ, BRCA, LUSC, UVM, pancreatic adenocarcinoma (PAAD), KIRC, as well as prostate adenocarcinoma (PRAD). Conversely, a negative correlation was observed in LAML and PCPG (**Figure 6A**), which aligns with the general BCAP31 expression pattern observed in various cancer types. Additionally, a relationship was found to be positive between BCAP31 expression as well as CNV in ESCA, as seen in **Figure 6B**. The gene mutation data for each kind of tumor was acquired from the UCSC Xena database. Subsequently, the R package “maftools” was used to generate visual representations of gene mutations within high and low expression categories. In the context of ESCA, the analysis revealed that the top ten genes that possessed the highest rates of mutation were TP53, DNAH5, FLG, CNTNAP5, TTN, CSMD3, MUC4, RYR2, MUC16, and SYNE1 in the BCAP31 high group. Conversely, the BCAP31 low group exhibited TP53, TIN, CSMD3, SYNE1, MUC16, FLG, MUC4, DNAH5, PCLO, and SPTA1 as the top ten genes (**Figures 6C, D**). Furthermore, the genes that exhibited variations in expression levels between the ESCA groups with high and low expression were identified as SDK1, CHD3, DCDC1, CNTNAP5, FCRL3, and NETO1, as seen in **Figure 6E**.

### GSVA analysis

**Figure 7:**
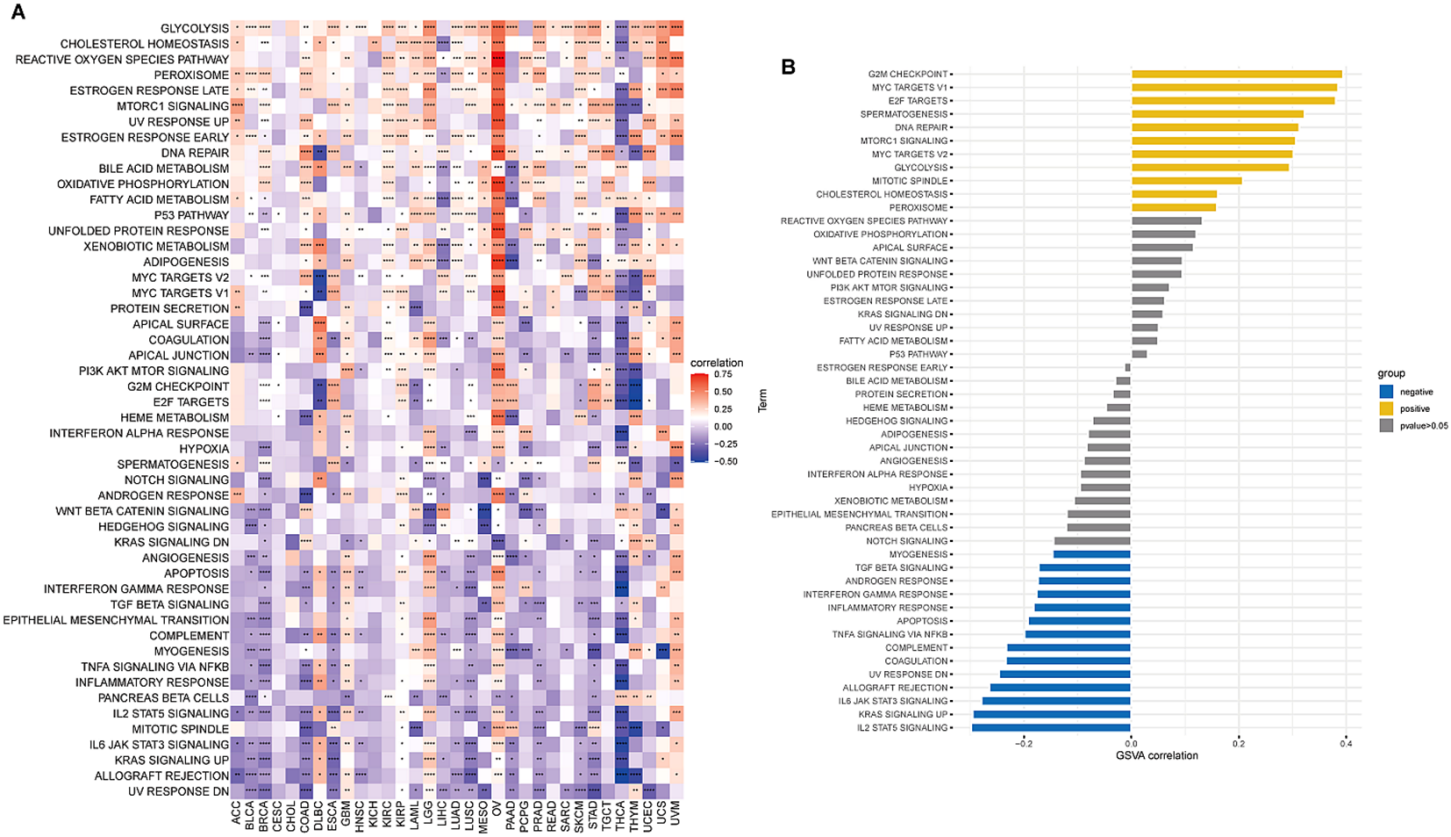
Results of GSVA (Gene Set Variation Analysis) for BCAP31 in pan-cancer and ESCA. **(A)** Heatmap showing GSVA enrichment of BCAP31 across various cancers, with red indicating a positive correlation and blue indicating a negative correlation. The color gradient ranges from purple (-0.5) to white (0) to red (0.75), representing the strength and direction of correlation between BCAP31 expression and pathway enrichment across different cancer types. **(B)** Bar plot displaying GSVA enrichment analysis results of BCAP31 specifically in ESCA (Esophageal Carcinoma), with pathways grouped by positive (yellow) and negative (blue) correlations, and gray indicating non-significant results (p-value > 0.05). *p < 0.05, **p < 0.01, ***p < 0.001, ****p < 0.0001.

To examine the possible involvement of BCAP31 in cancer, utilizing the GSVA was crucial to assess the associations between its gene expression and several biological signature pathways. In the context of GSVA, our findings indicate a significant association between BCAP31 and many pathways connected to proliferation and immunological response. These pathways include KRAS signaling up, PI3K AKT MTOR signaling, mTORC1 signaling route, TGF-β signaling, and IL-2 signaling pathway, as seen in **Figure 7A**. Furthermore, within the TCGA-ESCA cohort, there exists a positive correlation between the BCAP31 gene expression as well as the scores associated with various pathways. These pathways include G2M checkpoint, mTORC1 signaling, E2F targets, spermatogenesis, mitotic spindle, MYC targets v1, DNA repair, MYC targets v2, glycolysis, cholesterol, and homeostasis. Conversely, BCAP31 gene expression negatively correlates with the scores of the following pathways: UV response DN, myogenesis, TGF-beta signaling, inflammatory response, coagulation, androgen response, interferon gamma response, apoptosis, TNFA signaling via NFKB, KRAS signaling up, complement, allograft rejection, IL6 JAK STAT3 signaling, and IL2 STAT5 signaling (**Figure 7B**). The activation of tumor-related pathways, including those associated with the immune system, may contribute to worse patient outcomes in individuals with malignancies.

### ESTIMATE analysis

**Figure 8:**
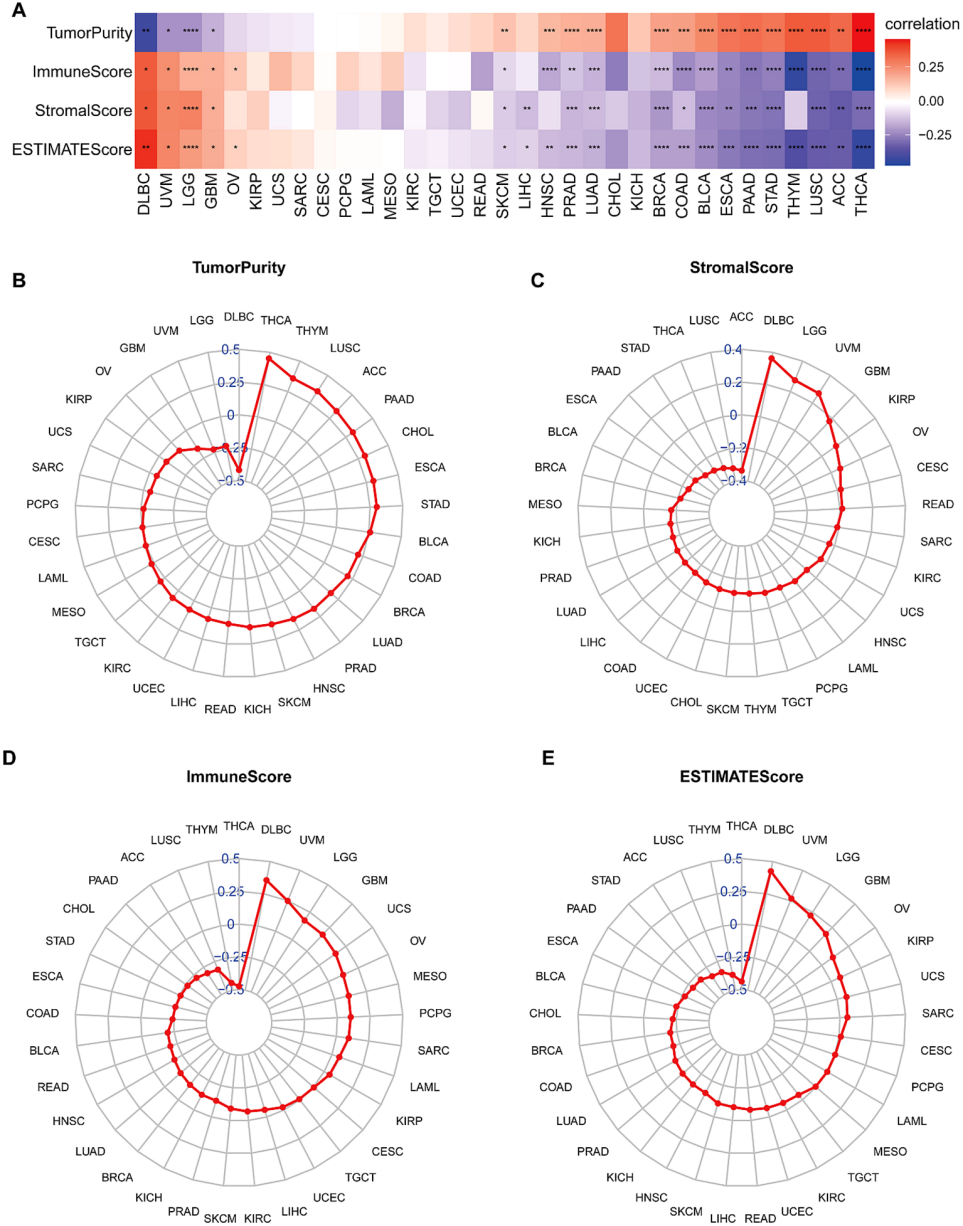
Correlation between BCAP31 expression and tumor microenvironment scores in pan-cancer analysis. **(A)** Heatmap showing the correlation between BCAP31 expression and TumorPurity, ImmuneScore, StromalScore, and ESTIMATEScore across various cancers, with a color gradient from purple (-0.25) to white (0) to red (0.25) indicating the correlation strength and direction. **(B)** Radar plot illustrating BCAP31’s correlation with TumorPurity in different cancers; **(C)** Radar plot of StromalScore correlations; **(D)** Radar plot of ImmuneScore correlations; **(E)** Radar plot of ESTIMATEScore correlations. *p < 0.05, **p < 0.01, ***p < 0.001, ****p < 0.0001.

The behavior of cancer cells is significantly influenced by the surrounding cells within the tumor microenvironment (TME). Our analysis examined the relationship between BCAP31 expression and various factors, including stromal score, ESTIMATE score, immune score, and tumor purity. The results showed a negative correlation between BCAP31 expression and tumor purity in patients with DLBC, UVM, LGG, and GBM (**Figure 8A**). A positive correlation was observed between BCAP31 expression and immune scores in DLBC, UVM, LGG, GBM, and OV. Moreover, there was a significant correlation between BCAP31 expression and stromal scores, except in cancers such as OV, KIRP, UCS, SARC, CESC, PCPG, LAML, MESO, KIRC, TGCT, UCEC, READ, HNSC, CHOL, KICH, and THYM. Similarly, BCAP31 expression showed a significant correlation with ESTIMATE scores across several cancer types, including DLBC, UVM, LGG, GBM, and OV. Additionally, we calculated TME-related scores, including stromal and immune scores, as well as tumor purity, as illustrated in **Figures 8B–E**. These findings suggest that BCAP31 plays a critical role in the disruption of the TME, affecting both stromal and immune components.

### TME analysis

**Figure 9:**
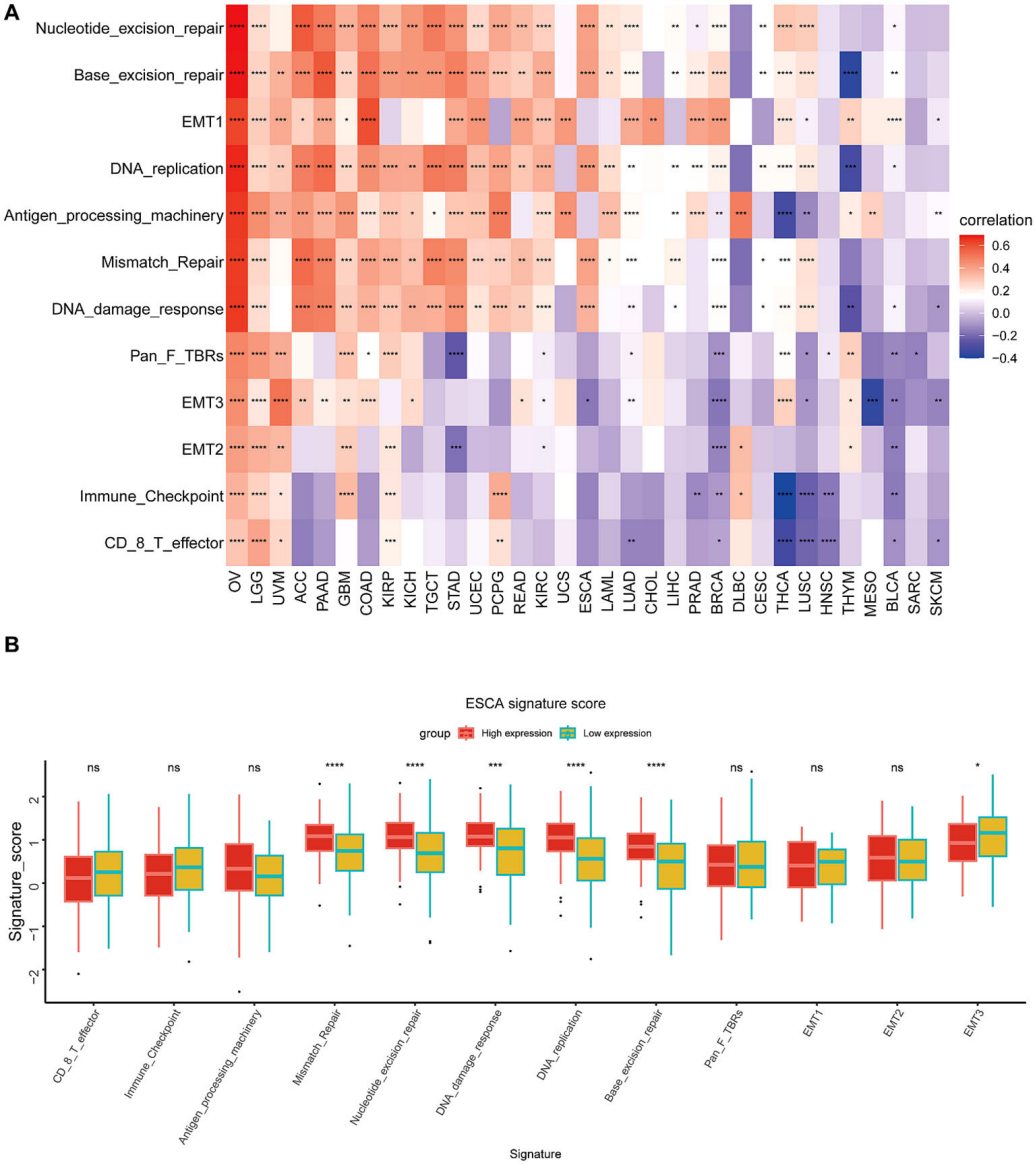
Tumor Microenvironment (TME) analysis of BCAP31 expression. **(A)** Heatmap showing the correlation between BCAP31 expression and various TME-related signatures across multiple cancer types. The color gradient ranges from purple (-0.4) to white (0) to red (0.6), representing the strength and direction of correlation between BCAP31 expression and TME components. **(B)** Boxplot showing the differences in TME signature scores between high and low BCAP31 expression groups in ESCA (Esophageal Carcinoma), with high expression in red and low expression in green. *p < 0.05, **p < 0.01, ***p < 0.001, ****p < 0.0001.

This particular research’s outcomes illustrated a correlation that is positive between BCAP31 as well as the TME in several cancer types, including OV, LGG, UVM, PAAD, GBM, COAD, KIRP, KICH, TGCT, UCEC, and PCPG. However, an inverse correlation was seen between BCAP31 and TME in HNSC, BLCA, SARC, and SKCM (**Figure 9A**). Furthermore, it is crucial to highlight the differences in the TME between the high and low expression cohorts of BCAP31 in ESCA. In particular, the analysis reveals that DNA replication, mismatch repair, DNA damage response, base excision repair, and nucleotide excision repair signatures are significantly associated with the elevated expression observed in the BCAP31 high-expression group (**Figure 9B**).

### TIMER2.0 analysis

**Figure 10:**
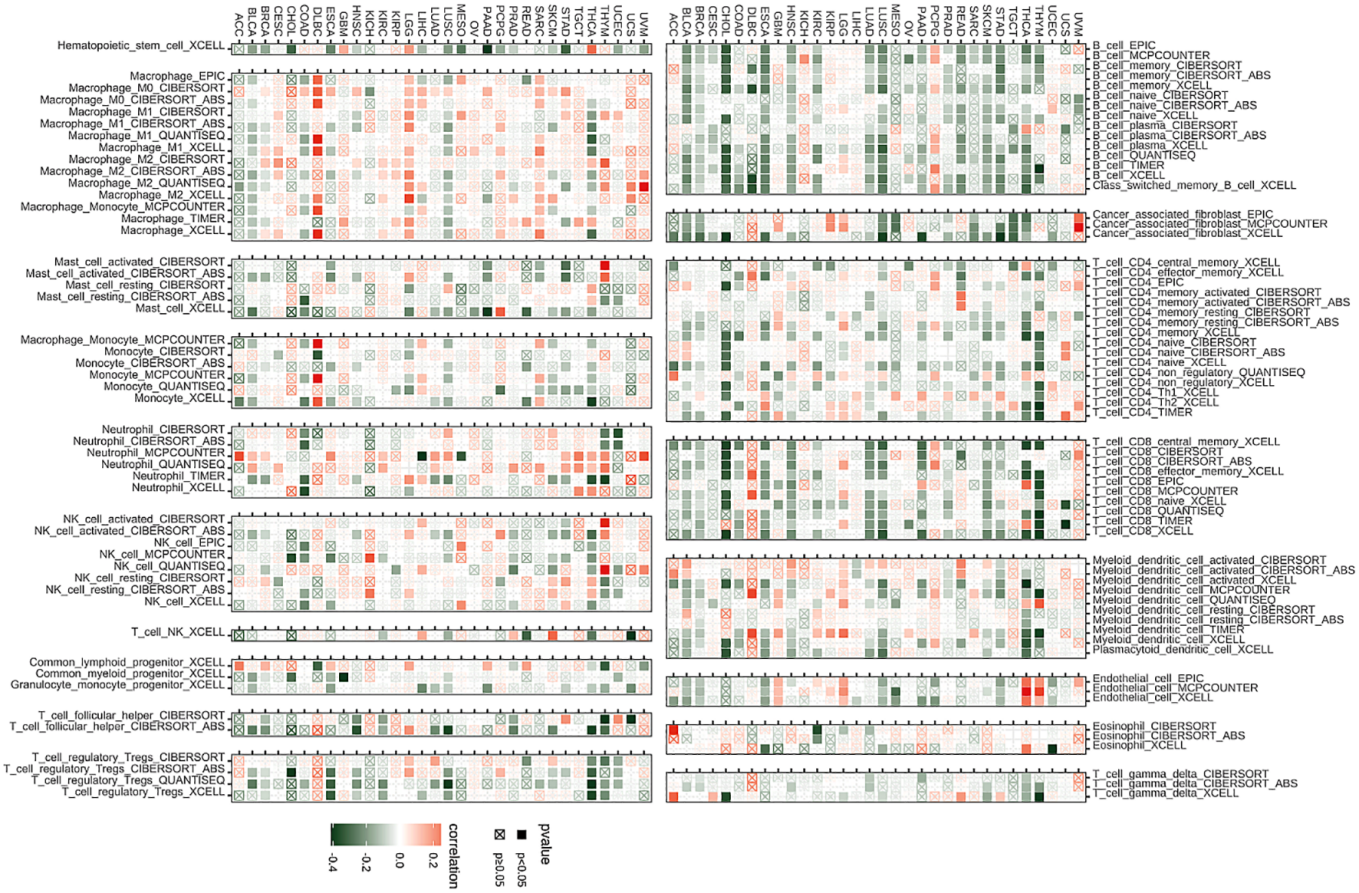
Immune infiltration analysis. Correlation analysis between BCAP31 expression and immune cell infiltration across various cancer types, using data from the TIMER2 database. Red squares indicate a positive correlation, green squares indicate a negative correlation, and white squares indicate no significant correlation. The color gradient ranges from green (-0.4) to white (0) to red (0.2), representing the strength and direction of correlation. Black-bordered squares represent significant correlations with p < 0.05, while crossed squares (box with an “X”) represent non-significant correlations with p ≥ 0.05.

The algorithms of TIMER2 were employed to assess the possible correlation between differences in the infiltration of different types of immune cells as well as the BCAP31 gene expression. In many types of tumors, BCAP31 expression shows a significant positive correlation with certain immune cells, such as myeloid cells, macrophages, neutrophils, and NK cells. Conversely, BCAP31 expression exhibits a significant negative correlation with other immune cells, including B cells, CD8+ T cells, regulatory T cells (Tregs), and follicular helper T cells. There is also a positive correlation between some tumors and cancer-associated fibroblasts (CAFs) (**Figure 10**).

### ImmueCellAI analysis

**Figure 11:**
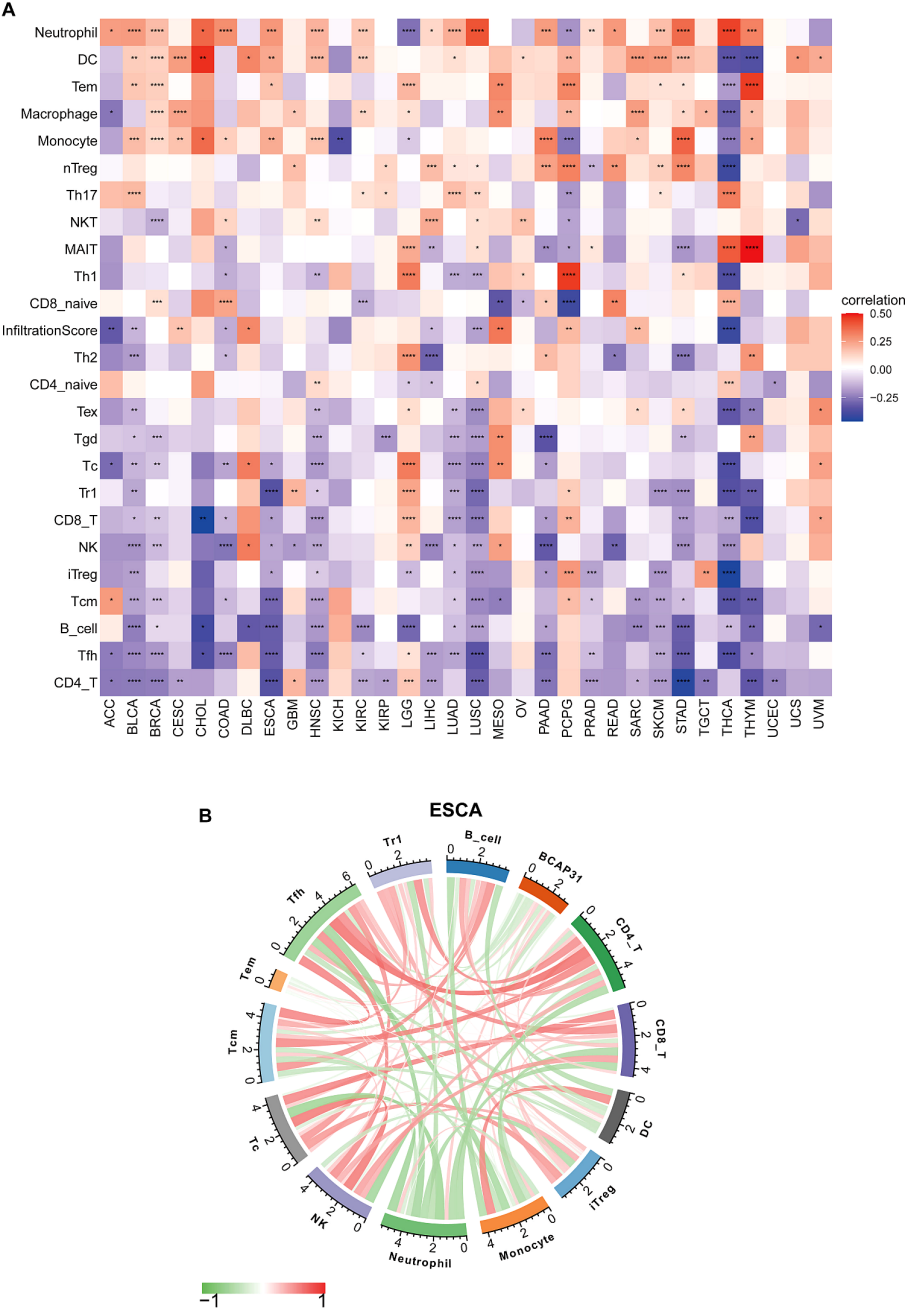
Immune cell infiltration analysis of BCAP31 expression. **(A)** Heatmap showing the correlation between BCAP31 expression and immune cell infiltration levels across various cancers using the ImmuCellAI database. The color gradient ranges from purple (-0.25) to white (0) to red (0.50), representing the correlation strength and direction. **(B)** Circos plot displaying immune cell infiltration analysis results specifically for BCAP31 in ESCA (Esophageal Carcinoma), with the color gradient ranging from green (-1) to red (1) indicating correlation values. *p < 0.05, **p < 0.01, ***p < 0.001, ****p < 0.0001.

Emerging studies have shown that tumor-infiltrating immunocytes might possess a significant influence on the OS outcomes of individuals. In this perspective, an examination was conducted on the relationships between the levels with regards to BCAP31 expression as well as the abundance of 25 different subtypes of immune cells that infiltrate at a pan-cancer level. The analysis was performed utilizing the ImmueCellAI database. Based on an analysis of data obtained from the ImmuCellAI database, results that we have obtained indicate a substantial positive correlation between BACP31 as well as various immune cell types, including Neutrophil, DC, Tem, macrophage, monocytes, nTreg, Th17, and NKT. In contrast, a correlation that is negative was discovered between BACP31 as well as Tgd, Tc, Tr1, CD8 T, NK, iTreg, Tcm, B cell, Tfh, and CD4 T (**Figure 11A**). These results suggest that BCAP31 may exert an immunosuppressive effect. In addition, we investigated the relationship between BCAP31 as well as immune cells in the TCGA-ESCA cohort. Our findings revealed a strong positive connection between BCAP31 and immune cell subsets such as Tem, neutrophil, monocyte, and DC. Conversely, BCAP31 displayed a negative correlation with B cell, Tr1, Tfh, Tcm, Tc, NK, iTreg, CD8 T, and CD4 T subsets (**Figure 11B**).

### Immune-related genes analysis co-expression analysis

**Figure 12:**
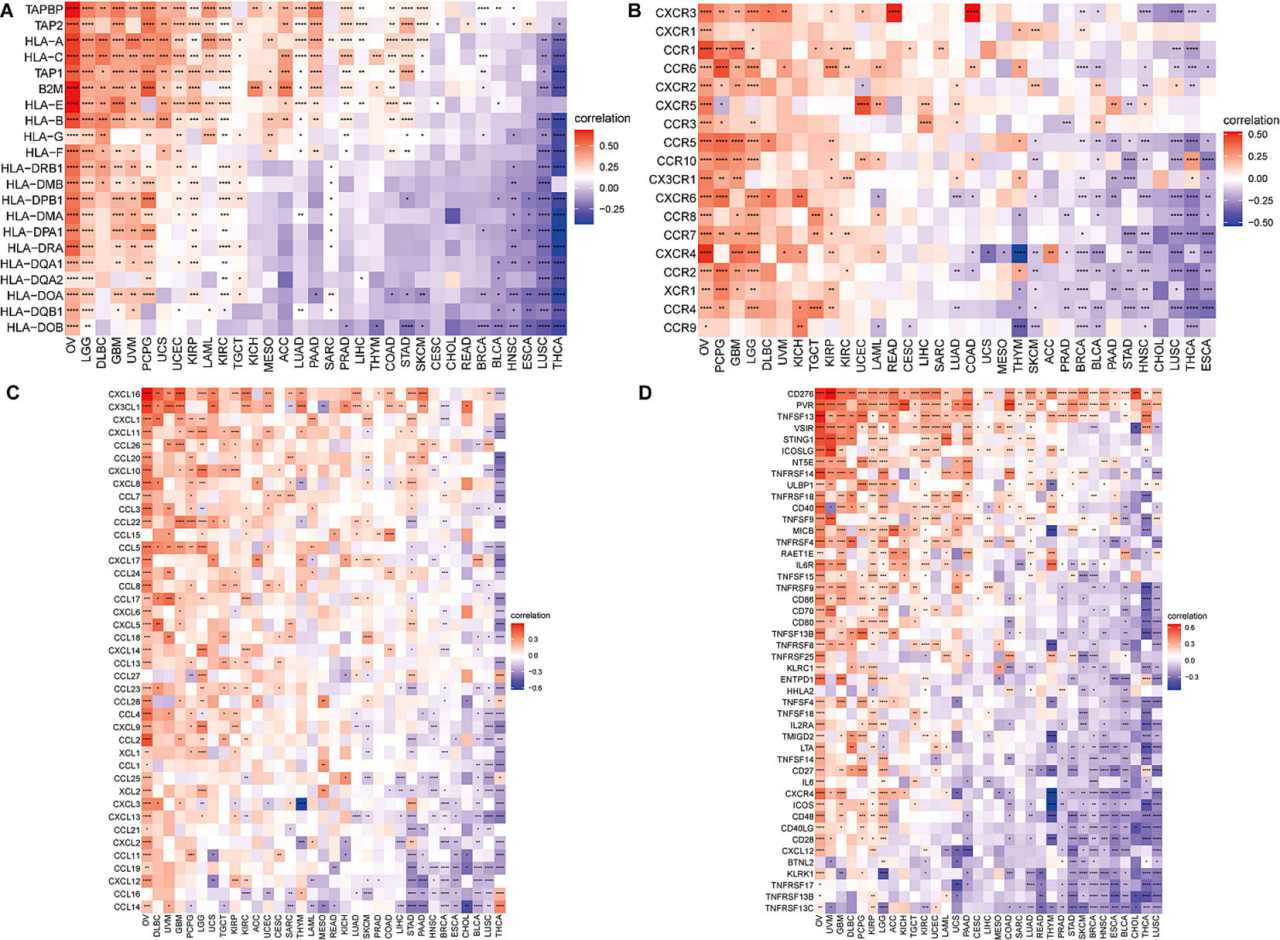
Correlation between BCAP31 and immune-related genes. **(A)** Heatmap showing the correlation between BCAP31 expression and MHC genes across various cancers, with a color gradient from purple (-0.25) to white (0) to red (0.50). **(B)** Correlation between BCAP31 and chemokine receptor genes, with the color gradient ranging from purple (-0.50) to white (0) to red (0.50). **(C)** Correlation between BCAP31 and chemokine genes, with a color gradient from purple (-0.60) to white (0) to red (0.30). **(D)** Correlation between BCAP31 and immune-activating genes, with the color gradient ranging from purple (-0.30) to white (0) to red (0.60). *p < 0.05, **p < 0.01, ***p < 0.001, ****p < 0.0001.

To attempt to get a deeper understanding of the regulatory mechanism behind tumor infiltration associated with BCAP31, this work examined the relationships between BCAP31 and four specific categories of immunomodulators: MHC genes, chemokine-receptor genes, chemokine genes, as well as immune-activating genes. These associations were analyzed using datasets obtained from TCGA. In addition, we performed gene co-expression studies in order to examine the associations between the BCAP31 expression as well as immune-related genes in a sample of 33 malignancies. The heatmap generated from the analysis revealed a significant co-expression between BCAP31 and almost all genes associated with the immune system. Furthermore, it has also been validated that the BCAP31 expression indicated a correlation that is positive with immunomodulators in OV, PCPG, LGG, KIRP, DLBC, GBM, UCEC, and UVM. Conversely, BCAP31 indicated a negative correlation with immunomodulators in ESCA, THCA, LUSC, CHOL, HNSC, BRCA, and STAD (**Figures 12A–D**).

### Drug sensitivity analysis

**Figure 13:**
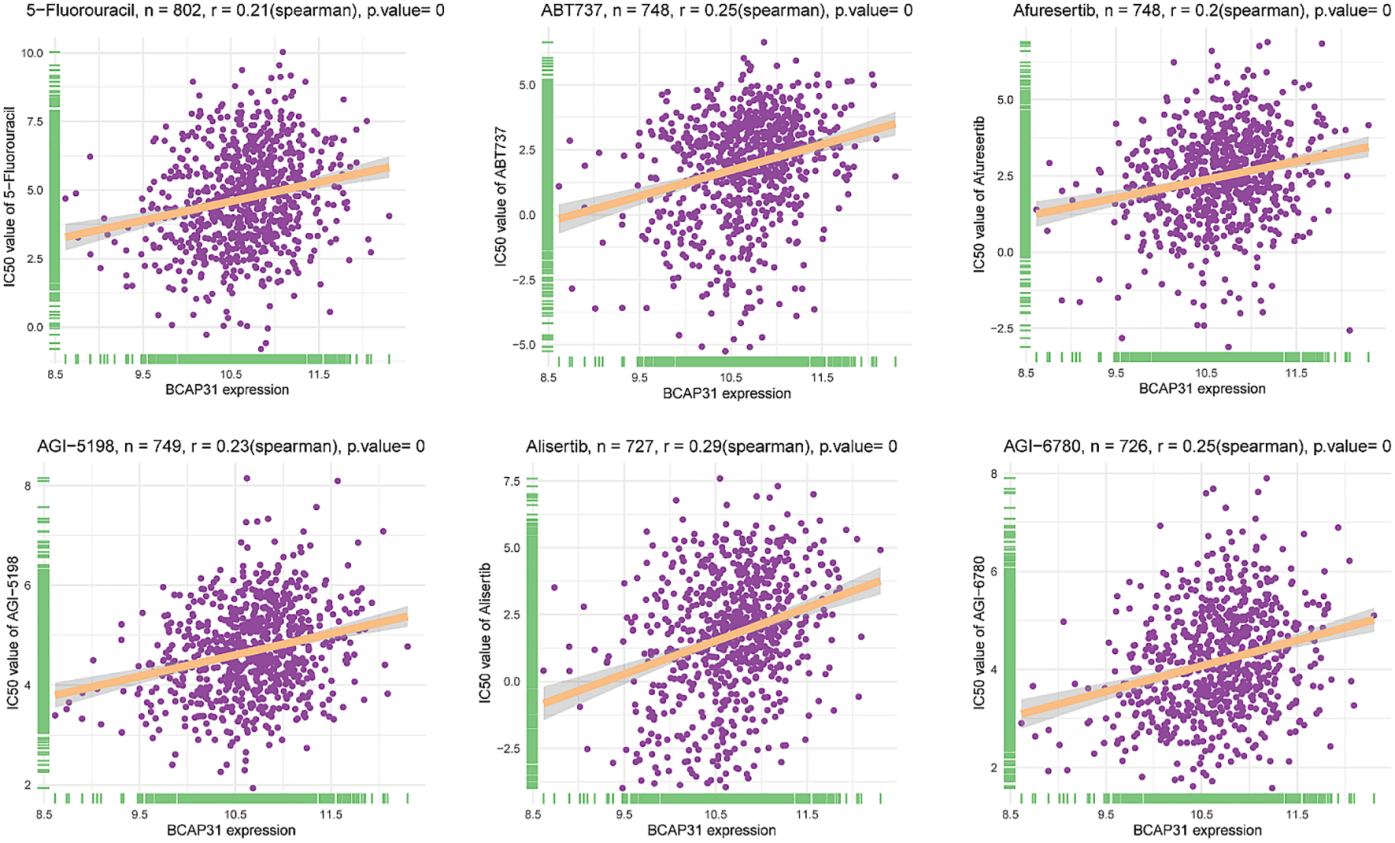
Correlation analysis between BCAP31 expression and drug sensitivity (IC50 values) in various compounds. Scatter plots illustrate the correlation between BCAP31 expression levels and IC50 values (drug sensitivity) for different compounds: 5-Fluorouracil (n = 802, r = 0.21, p < 0.001); ABT737 (n = 748, r = 0.25, p < 0.001); Afuresertib (n = 748, r = 0.20, p < 0.001); AGI-5198 (n = 749, r = 0.23, p < 0.001); Alisertib (n = 727, r = 0.29, p < 0.001); AGI-6780 (n = 726, r = 0.25, p < 0.001). Each plot shows a positive correlation between BCAP31 expression and drug IC50, suggesting that higher BCAP31 expression may be associated with increased resistance to these compounds. The Spearman correlation coefficient and p-value are indicated for each analysis.

A comprehensive set of 198 pharmaceutical compounds has been discovered to have an association with BCAP31. The present study demonstrated the six medicines that had the most robust positive connection. The medicines shown to have a positive connection with BCAP31 include 5-Fluorouracil (R = 0.21), ABT737 (R = 0.25), Afuresertib (R = 0.2), AGI-5198 (R = 0.23), Alisertib (R = 0.29), and AGl-6780 (R = 0.25) (**Figures 13A–F**).

### Immunotherapy analysis

**Figure 14:**
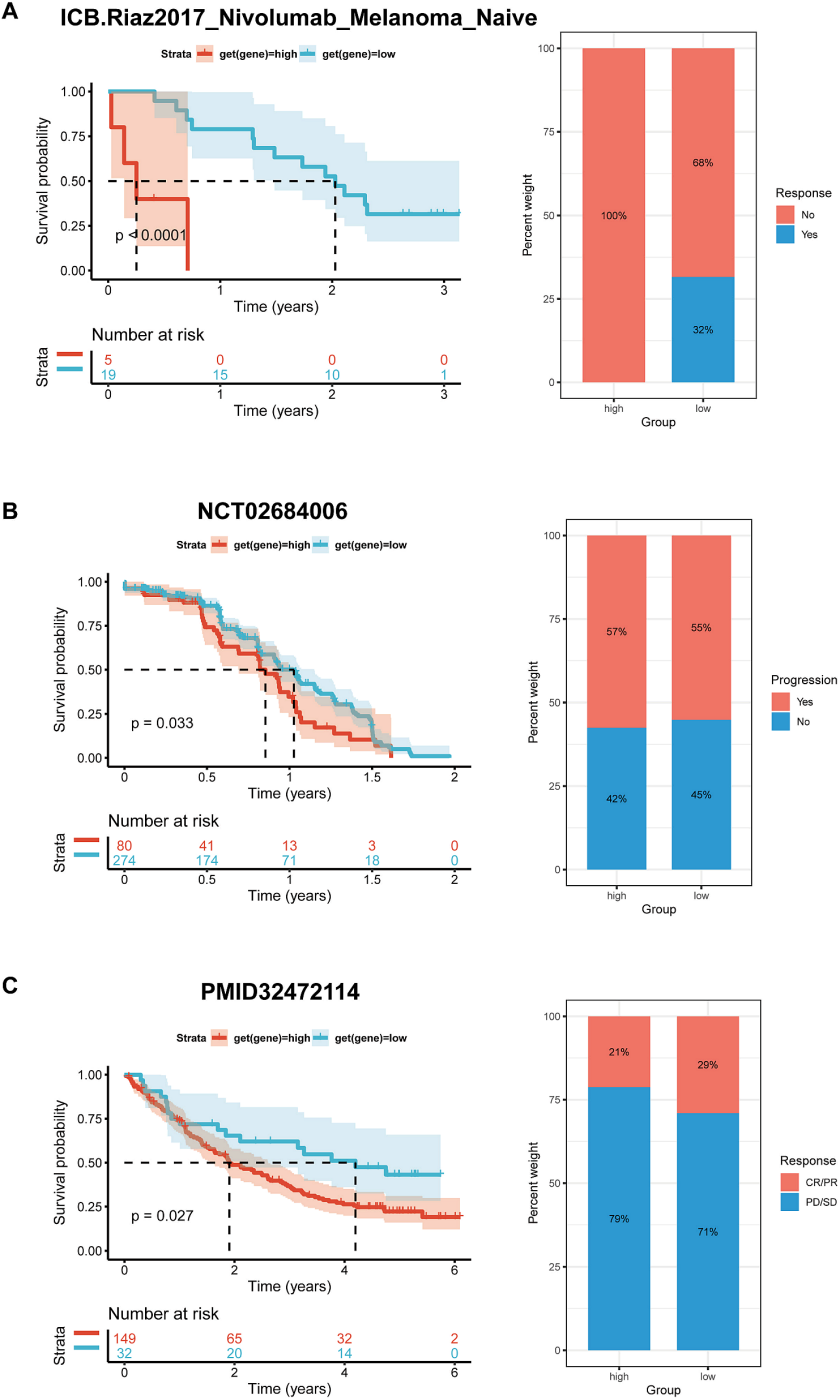
Correlation between BCAP31 expression and immunotherapy response. **(A)** Survival analysis and treatment response proportion in high and low BCAP31 expression groups in the ICB Riaz2017 Nivolumab Melanoma dataset, showing significant survival differences (p < 0.0001) and response rates with 100% non-responders in the high group vs. 68% in the low group. **(B)** Survival analysis and treatment response proportion in high and low expression groups in the NCT02684006 dataset, with a significant difference in survival (p = 0.033) and response rates (57% progression in high vs. 55% in low). **(C)** Survival analysis and treatment response proportion in high and low expression groups, showing significant survival differences (p = 0.027) and response rates with 21% responders in the high group vs. 29% in the low group. The left panels display Kaplan-Meier survival curves, and the right panels show bar charts representing the response proportions for each group.

Preliminary evidence suggests that BCAP31 has a significant impact on the immune cell infiltration in the TMB. Meanwhile, the efficacy of immunotherapy is directly linked with immunological checkpoint expressions on the surface of immune cells. To gain in-depth knowledge with respect to the possible correlation between the expression level of BCAP31 and the prognosis of tumor patients undergoing immunotherapy, we analyzed it in order to examine the variances in therapeutic response and prognosis across various subgroups of BCAP31 expression. This analysis was performed on three cohorts of patients who were receiving immunotherapy for melanoma (**Figure 14A**) and advanced renal cell carcinoma (**Figures 14B, C**). In accordance with our anticipated findings, a substantial correlation was discovered between elevated levels regarding BCAP31 expression. It diminished therapeutic response as well as worse outcomes among individuals with tumors who underwent immunotherapy.

### Function assay

**Figure 15:**
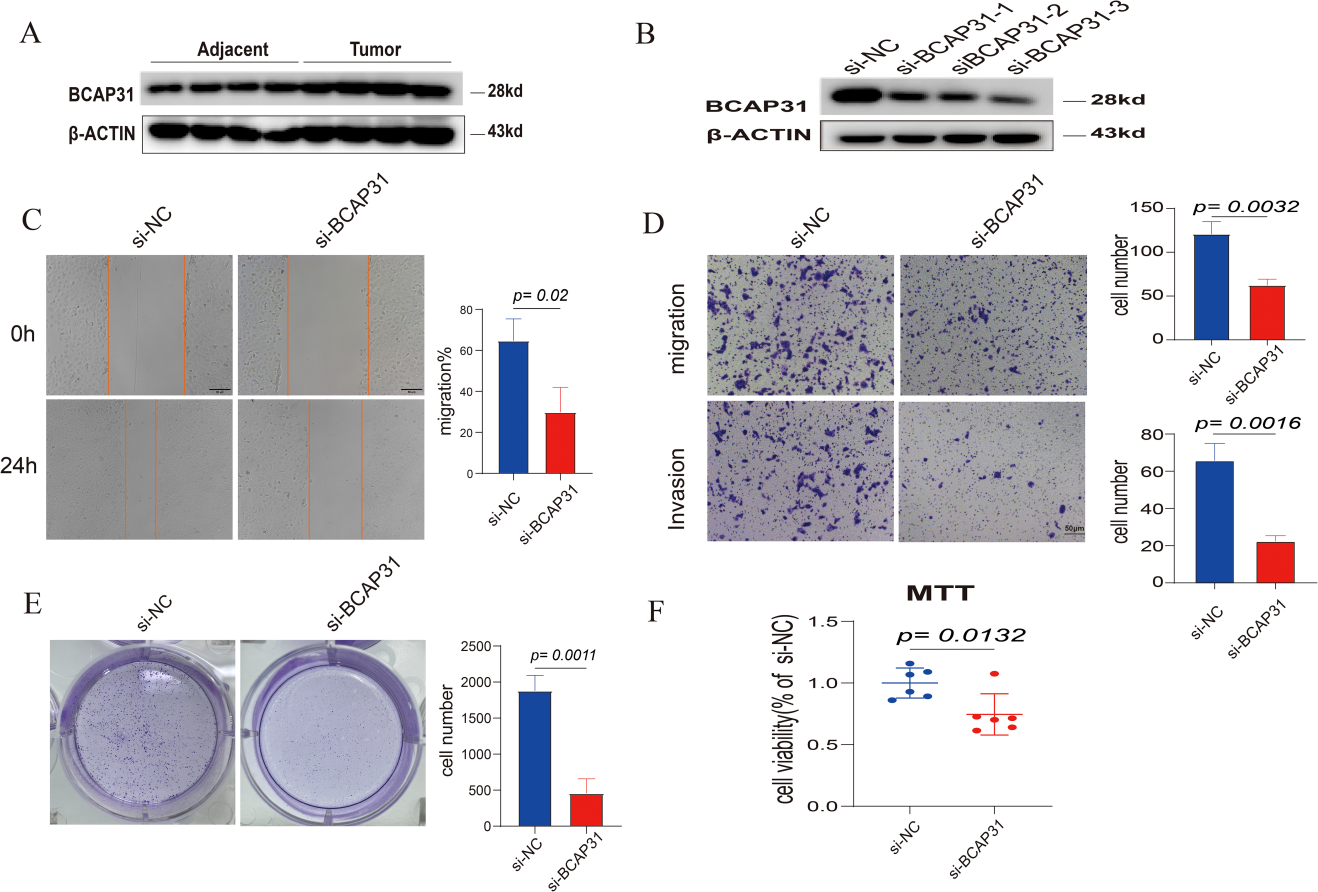
Impact of BCAP31 knockdown on KYSE-150 cell functions. **(A)** Western blot analysis showing increased BCAP31 expression in four paired adjacent and tumor tissues, consistent with bioinformatics results. **(B)** Confirmation of efficient BCAP31 downregulation in KYSE-150 cells using siRNA, as evidenced by Western blot analysis. **(C)** Wound healing assay showing reduced migration in BCAP31 knockdown cells compared to control (si-NC), with quantification (p = 0.02). **(D)** Transwell assays demonstrating decreased migration and invasion abilities in BCAP31 knockdown cells compared to control, with quantification (migration: p = 0.0032; invasion: p = 0.0016). **(E)** Colony formation assay showing a significant reduction in colony-forming ability in BCAP31 knockdown cells (p = 0.0011). **(F)** MTT assay indicating decreased cell viability in BCAP31 knockdown cells compared to control (p = 0.0132).

WB analysis showed that BCAP31 was increased in four paired tissues, which was similar to the abovementioned bioinformatic result (**Figure 15A**). To explore the functions of BCAP31 in ESCA, we utilized a siRNA to reduce the BCAP31 expression levels in KYSE-150 cells. WB analysis showed that this siRNA could downregulate the expression of BCAP31 in the cell lines with high efficiency (**Figure 15B**). As a functional assay, transwell, as well as wound healing assays, were utilized to assess the impact of BCAP31 on the migration along with the metastatic capacity of KYSE-150 cells. Here, the findings demonstrated that migration as well as invasion with respect to BCAP31-downregulated KYSE-150 cells was significantly faster than the control group infected with si-BCAP31 (**Figures 15C, D**). It suggests that *in-vitro* migration and invasion of KYSE-150 cells were inhibited by BCAP31. Furthermore, BCAP31 knockdown can significantly promote the colony formation abilities of KYSE-150 cells (**Figure 15E**). On the other hand, the proliferation rate of KYSE-150 cells was measured using the MTT assay following BCAP31 knockdown using either the control or si-BCAP31. The BCAP31 knockdown group’s cell viability and quantity were significantly lower than the control group, as per the findings (**Figure 15F**). Furthermore, five pairs of tumor and adjacent normal tissues from each of the three types of cancers (ESCA, LUAD and GA) exhibited higher BCAP31 expression levels in the tumor tissues compared to the adjacent normal tissues, as confirmed by IHC results (**Supplementary Figures S1**–**S3**).

## Discussion

Initially, an evaluation was conducted to determine the mRNA expression levels of BCAP31 within tumor samples. Consistent with prior research, Mwichie et al. observed that BCAP31 was shown to be overexpressed in several malignant tissues based on the HPA pathology datasets (27). Additionally, our findings demonstrated a considerable up-regulation of BCAP31 expression in the majority of cancer cases. Moreover, through IHC, we discovered that the expression of BCAP31 increased in ESCA, GA, and LUAD. Nevertheless, it was shown that the expression pattern of this gene had an inverse tendency in patients with LAML and DLBC, suggesting its potential role as a dual-action factor in the growth of tumors. Moreover, to examine the possible correlation between the BCAP31 expression level as well as patient survival, a comprehensive analysis of the cancer patients’ prognosis in relation to BCAP31 expression levels across all cancer types was conducted utilizing TCGA datasets. K-M survival curves were created, and an analysis using the Cox proportional hazards model was performed to identify the prognostic significance of BCAP31 in various tumor types. The presence of elevated levels of BCAP31 was shown to be indicative of an adverse prognosis in many types of cancers, including ESCA, LAML, CESC, BRCA, COAD, HNSC, GBM, KIRP, LGG, LUAD, and SKCM. On the other hand, a decreased expression of BCAP31 in colorectal cancer (CRC) tissues is indicative of a worse prognosis after surgical intervention (12). In hepatocellular carcinoma, there exists a correlation between low BCAP31 expression as well as a negative prognosis after surgical resection (13). Moreover, our investigation revealed a substantial correlation between the BCAP31 expression levels as well as the clinical stages of different forms of malignancies. This observation indicates that BCAP31 might potentially serve an essential function in informing the clinical management of cancer patients across various tumor pathological stages. In summary, our work has provided evidence to support the significant involvement of BCAP31 in the development of tumors, hence establishing its potential as a prognostic predictor.

To investigate the role of BCAP31, we conducted a GSVA analysis. It was shown that many prevalent pathways associated with cancer, such as DNA repair and mTORC1 signaling pathway, exhibited enrichment in several cancer types characterized by elevated levels of BCAP31. Additionally, a significant decrease in the expression of proteins associated with the cell cycle has been seen (6). BCAP31 has been shown to have a substantial role in the advancement of non-small cell lung cancer (NSCLC) by facilitating the migration as well as invasion with regard to NSCLC cells via the Akt/mTOR/p70S6K pathway (9). The reduction of BCAP31 is crucial to inhibiting cell proliferation in CRC cells. This inhibition occurs mechanistically via the signaling pathway involving emerin and β-catenin (28). Furthermore, our experimental research results indicate that downregulating the expression of BACP31 in ESCA could inhibit cell proliferation and promote invasion as well as migration in KYSE-150 cells.

Simultaneously, it has been shown that BCAP31 plays a pivotal function in the immune system, not only facilitating the transportation of MHC molecules from the ER (29) but also contributing to the maturation and activation of T-cell antigen receptors (30). Impaired T-cell activity may prompt cancer cells to evade immune surveillance via many pathways. Cancer cells use a range of inherent immunological checkpoints inside T-cells in order to evade immune monitoring, making these pathways crucial for evasion (31). Furthermore, Niu et al. discovered that the regulatory function of BCAP31 in T-cell signaling is of considerable importance in the process of T-cell activation (32). The impact of BAP31 knockout in macrophages on CD4T cell activation is shown via the upregulation of MHC class II molecules (33). Moreover, prior research has demonstrated that BAP31 affects macrophage polarization via its regulation of helper T-cell activation (34). Consequently, an immunological study was conducted. In accordance with prior research, the outcomes we have obtained illustrate the T-cells activation has a role in the advancement of 33 various forms of cancer.

To carry out a more comprehensive examination of the influence of BCAP31 on tumor progression, we initiated a discussion on the involvement of BCAP31 in the TME. The TME consists of several biological components, including tumor cells, stromal cells, diverse immune cell populations, and the extracellular matrix. The TME is essential to the development of tumors as well as their response to therapy. Immune cells, as well as stromal cells’ non-tumor components, possess significant clinical importance in the diagnostic and prognostic evaluation of malignancies. BCAP31 had a positive association with immunological scores, stromal scores, and ESTIMATE ratings for the TME in the majority of human cancer types. These outcomes indicate a correlation between the expression levels of BCAP31 as well as the TME, which includes the immune cells’ influx of the tumor. In addition, the evaluation of immune cell infiltration into tumors is a crucial factor that is connected to immunotherapy’s clinical effectiveness (35). Our research’s outcomes discovered that BCAP31 possesses significance in the infiltration of immune cells into tumors across many forms of cancer. Moreover, the BCAP31 expression levels displayed a favorable association with the ESTIMATE scores in many forms of cancer. The ESTIMATE score has a negative correlation with tumor purity, as shown by previous research (25). The presence of a low tumor purity has been shown to be correlated with advanced cancer stages and a worse prognosis (36).

Moreover, CAFs have a substantial impact on the TME as stromal constituents that influence tumor behavior (37). These cells have a role in facilitating malignant cell invasion and migration by producing growth factors and cytokines, modifying the extracellular matrix and boosting angiogenesis. Furthermore, these factors have the potential to lead to the emergence of resistance to therapy as well as the recurrence of tumors (38, 39). Recent research has brought attention to the growing significance of CAFs in the control of the immune system. These CAFs have been shown to influence the recruitment of immune cells inside the TME and facilitate immune evasion. This finding is supported by other studies (40). Our study revealed a strong correlation between CAFs and high expression of BCAP31 in the following types of tumors: DLBC, GBM, KIPP, LGG, and UVM.

Moreover, genetic modifications result in abnormal gene expression and the development of cancer (41). CNVs are a significant category of genetic structural variations that are essential in the discipline of cancer diagnosis, prevention, and therapy. Moreover, it has been shown that gene alterations generated by CNV might potentially result in immune evasion (42). In the present investigation, a notable frequency of CNVs was detected in many cancer types, including SARC, THYM, CESC, HNSC, ESCA, STAD, LIHC, CHOL, BLCA, READ, LUSC, UVM, PAAD, BRCA, LUAD, KIRC, OV, and PRAD. Conversely, a lack of CNV occurrence was seen in LAML and PCP. These results suggest a possible function for CNVs in the development of carcinogenesis. Furthermore, tumor-infiltrating immune cells have been determined as crucial elements with regard to the TME, serving as indicators for both prognosis and responsiveness to immunotherapy across different types of carcinomas (28). Several indicators, such as immunological checkpoints and TMB, have been used to assess the potential efficacy of immunotherapy (29). The TP53 gene is responsible for encoding the tumor protein p53, a crucial tumor suppressor that is crucial in inhibiting cell division and proliferation (43). The TMB is widely regarded as a biomarker that may be utilized to anticipate the effectiveness of immune checkpoint inhibitors (44). The present investigation revealed a positive correlation between high-BCAP31 individuals and elevated TP53 mutation rates, which in turn were associated with a much worse prognosis for the patients.

The experimental results provide compelling evidence for BCAP31’s role in tumor progression, particularly in ESCA. Western blot analysis confirmed elevated BCAP31 expression in tumor tissues compared to normal adjacent tissues, corroborating previous bioinformatic findings. The knockdown of BCAP31 in KYSE-150 cells resulted in a marked reduction in migration and invasion capabilities, as shown by the Transwell and wound healing assays. These findings suggest that BCAP31 contributes to the metastatic potential of ESCA cells. Interestingly, BCAP31 knockdown also promoted colony formation, indicating its role in regulating cell proliferation and survival under *in vitro* conditions. Further supporting the tumor-promoting functions of BCAP31, the MTT assay demonstrated significantly reduced cell viability in BCAP31-silenced cells, reinforcing its involvement in cellular proliferation pathways. The observed higher BCAP31 expression in tumor tissues of ESCA, LUAD, and GA compared to corresponding normal tissues, as confirmed by immunohistochemistry, suggests that BCAP31 overexpression is a common feature across various malignancies. Taken together, these results indicate that BCAP31 plays a multifaceted role in tumor progression, influencing migration, invasion, and proliferation. BCAP31’s involvement in these processes suggests that it may serve as a potential therapeutic target, particularly for inhibiting metastasis in ESCA and other cancers where its expression is elevated. The positive correlation between BCAP31 expression and drug sensitivity to 5-Fluorouracil, ABT737, Afuresertib, AGI-5198, Alisertib, and AGI-6780 suggests that BCAP31 could serve as a predictive biomarker for the efficacy of these drugs in specific cancers. High BCAP31 expression may indicate increased sensitivity to 5-Fluorouracil due to BCAP31’s involvement in cellular stress responses, while its association with ABT737 (a Bcl-2 inhibitor) implies altered apoptosis pathways that could enhance drug response. Similarly, BCAP31’s role in ER stress and apoptosis may make tumors more susceptible to Afuresertib (an AKT inhibitor), as well as to AGI-5198 and AGI-6780, which target mutant IDH1 and IDH2 respectively and are potentially effective in BCAP31-high tumors due to their interaction with metabolic pathways. Furthermore, Alisertib, an Aurora kinase inhibitor, may be particularly effective in highly proliferative tumors with elevated BCAP31, given its role in supporting cell survival pathways. Assessing BCAP31 expression could thus help stratify patients likely to benefit from these therapies, guiding personalized treatment strategies that optimize therapeutic efficacy and reduce exposure to less effective treatments.

We believe this is the first pan-cancer BCAP31 investigation. This research thoroughly examined pan-cancer data from several databases. Our research has inherent limitations. First, in prior differentially expressed gene analyses, BCAP31 was not differently expressed across OV, PCPG, and SARC tumor tissue and normal tissue. Thus, BCAP31’s diagnostic and prognostic utility in OV, PCPG, and SARC should be confirmed with many patients. Second, as our research analyzes current data, further experimental investigations are required to confirm bioinformatics findings. Further study will explain BCAP31’s molecular involvement. Third, pan-cancer research showed that BCAP31 expression was associated with immunomodulatory processes as well as immune cell infiltration. However, the mechanism is unknown.

## Conclusion

The study demonstrates that BCAP31 expression is significantly elevated in various tumor types. This elevated expression is associated with tumor progression, immune infiltration, and poor prognosis in several cancers. Functional assays further confirm that BCAP31 plays a critical role in promoting tumor cell migration, invasion, and proliferation. These findings suggest that BCAP31 may serve as a potential biomarker for cancer prognosis and a target for therapeutic intervention.

## Data Availability

The original contributions presented in the study are included in the article/**Supplementary Material**. Further inquiries can be directed to the corresponding author.

## Supplementary material

Supplementary Figures S1-S3 Immunohistochemical analysis of BCAP31 expression. Immunohistochemical results demonstrating elevated BCAP31 expression in tumor tissues compared to adjacent normal tissues across five pairs of samples from each of the three cancer types: ESCA, LUAD and GAD.
