# Supplementary figures and images for: Pan-cancer analysis shows that BCAP31 is a potential prognostic and immunotherapeutic biomarker for multiple cancer types
Source: Front Immunol. 2024 Dec 16;15:1507375. doi: 10.3389/fimmu.2024.1507375 (PMC11683684; doi:10.3389/fimmu.2024.1507375)

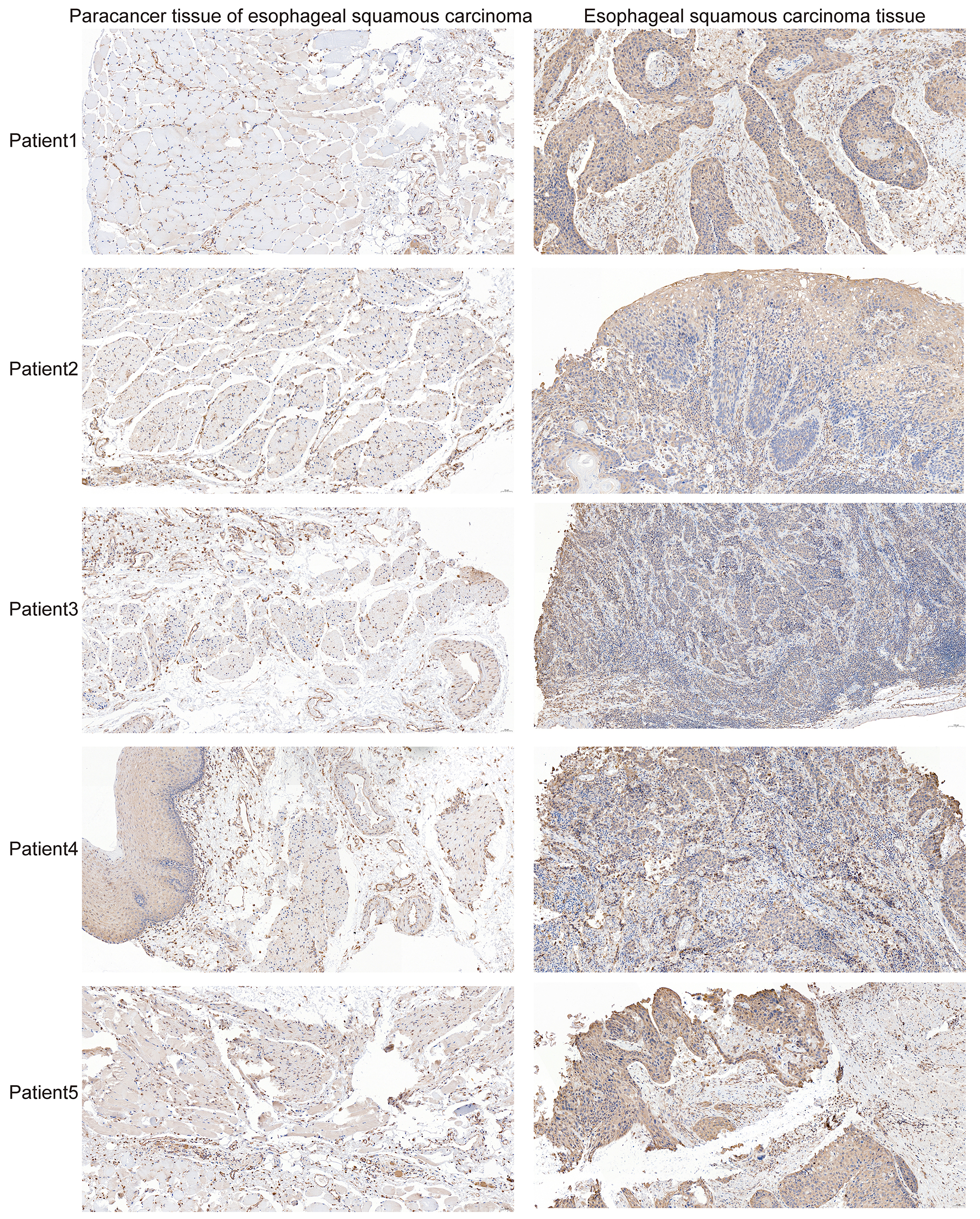

Supplement: Supplementary Figures S1-S3 — Immunohistochemical analysis of BCAP31 expression. Immunohistochemical results demonstrating elevated BCAP31 expression in tumor tissues compared to adjacent normal tissues across five pairs of samples from each of the three cancer types: ESCA, LUAD and GAD. [file Image1.jpeg]

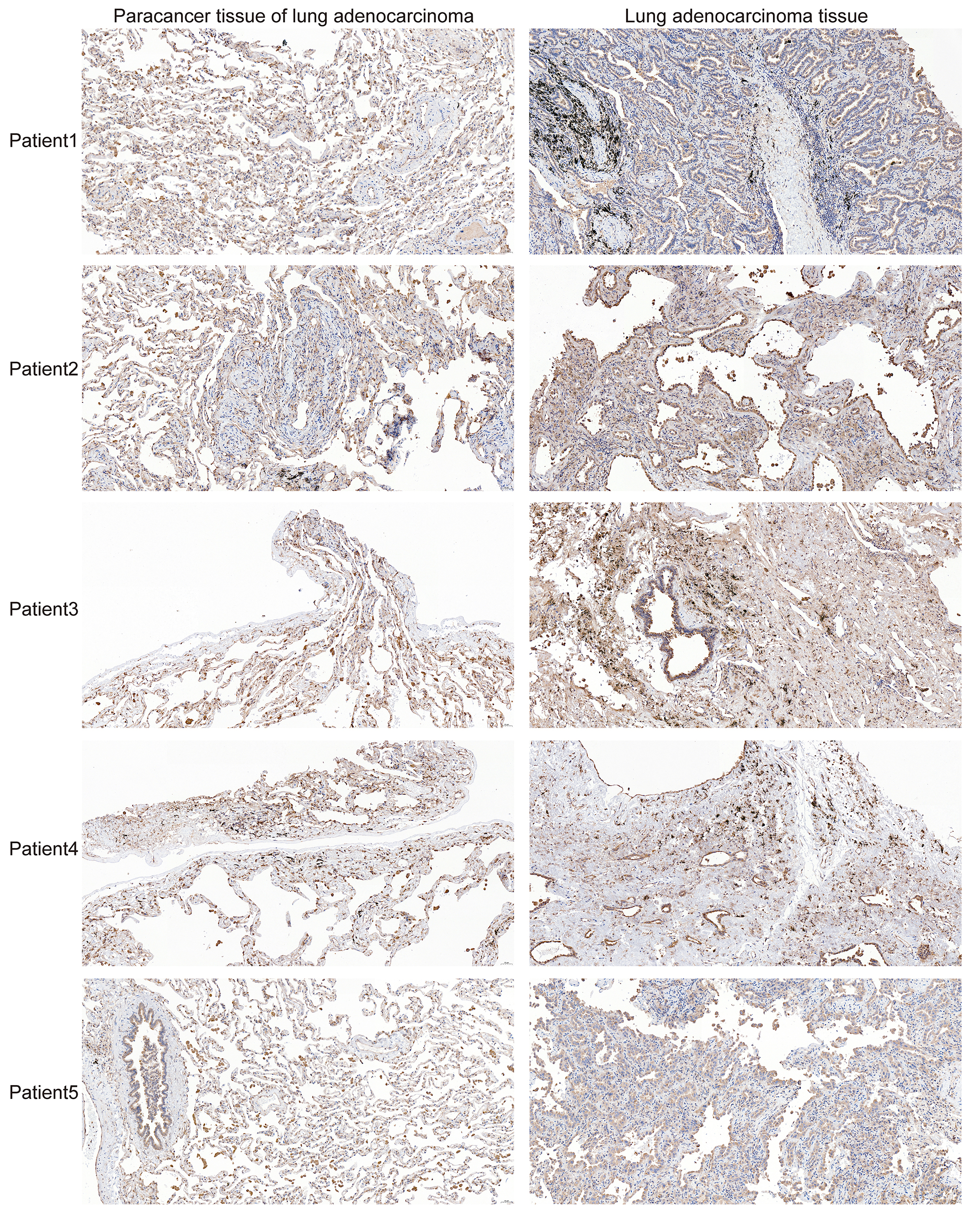

Supplement: Supplementary file 2 [file Image2.jpeg]

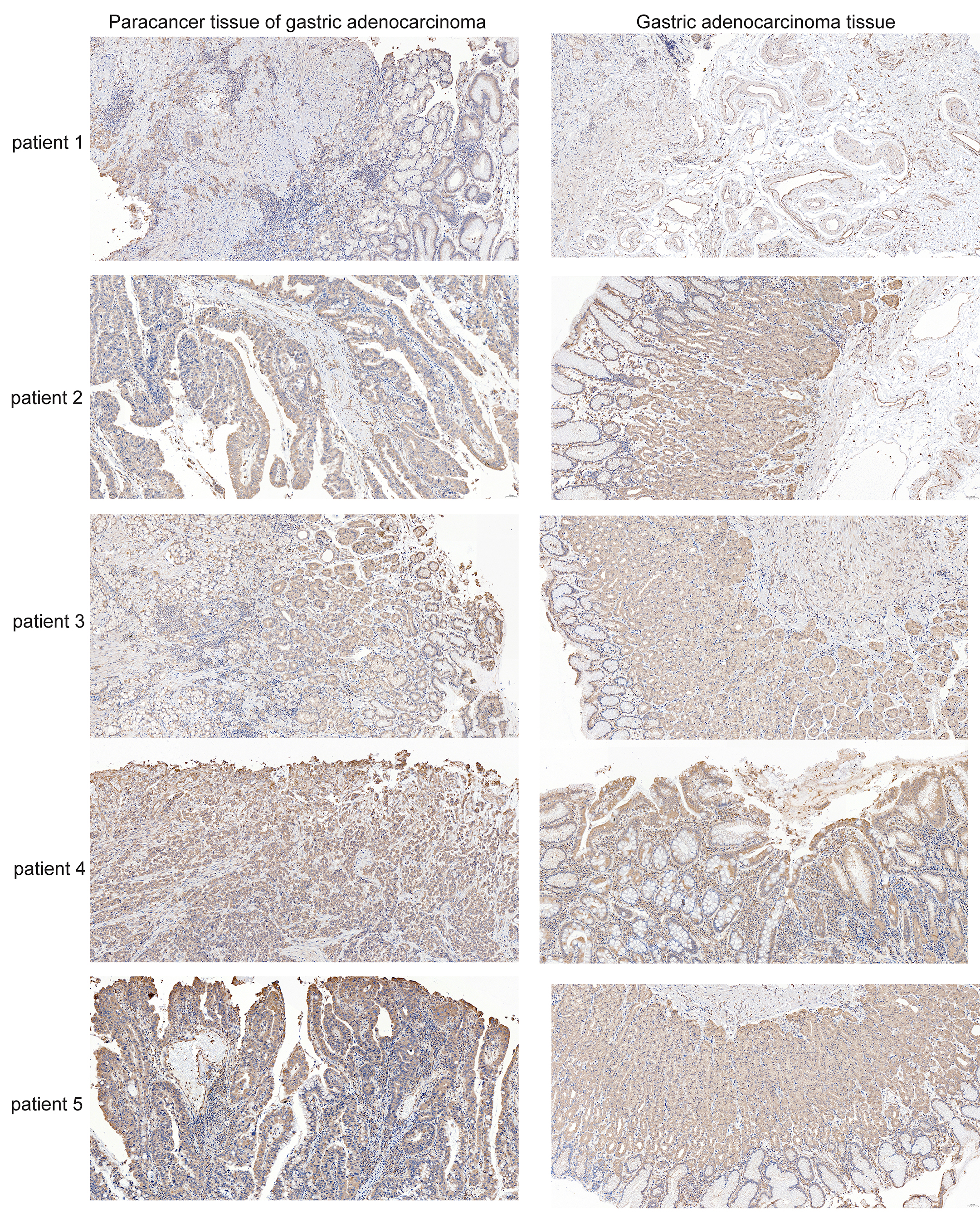

Supplement: Supplementary file 3 [file Image3.jpeg]
